# Bionic nanovesicles sequentially treat flaps with different durations of ischemia by thrombolysis and prevention of ischemia-reperfusion injury

**DOI:** 10.1016/j.mtbio.2025.101529

**Published:** 2025-01-30

**Authors:** Linzhong Yang, Yuanchen Liu, Cheng Tao, Zichen Cao, Shilin Guo, Zheng Wei, Yanyi Wang, Tao Liu, Lin Chen, Ke Xiong, Xingyu Luo, Jianchuan Ran, Wei Han

**Affiliations:** a Department of Oral and Maxillofacial Surgery, Nanjing Stomatological Hospital, Affiliated Hospital of Medical School, Institute of Stomatology, Nanjing University, Jiangsu, 210008, China; b Capital Medical University, Beijing Key Lab Tooth Regenerate & Function Reconstruct, Beijing Lab Oral Health, 10 You Men Wai Xi Tou Tiao, Beijing, 100069, China; c Pediatric Dentistry, Nanjing Stomatological Hospital, Affiliated Hospital of Medical School, Institute of Stomatology, Nanjing University, Nanjing, Jiangsu, 210008, China; d Department of Orthopedics, Taizhou People's Hospital of Nanjing Medical University, Taizhou School of Clinical Medicine, Nanjing Medical University, Taizhou, 225300, China

**Keywords:** Bionic nanovesicles, Flap repair, Ischemia-reperfusion injury, Thrombus-targeted, Flap survival time

## Abstract

Flap transplantation is a critical part of the recovery process for patients who have undergone tumor resection. However, the process of ischemia-reperfusion injury during flap transplantation and the resulting high-risk thrombotic microenvironment are unavoidable. In this study, based on an in-depth investigation of the ischemia time and prognosis of transplanted flaps, we propose a treatment strategy using sequential thrombolysis and ischemia-reperfusion injury prevention tailored to the ischemia time. This approach is designed to minimize the likelihood of thrombus formation and to clear the intravascular inflammatory microenvironment, with the aim of preventing and salvaging ischemic flaps. Specifically, we have successfully constructed a clinical-grade bionic vesicle, UK-PBNZ@PM, a system that cleverly incorporates drug components that have been widely used in clinical applications, thereby demonstrating a high degree of clinical translational potential. Prussian blue nano-enzymes (PBNZ) are the core component and demonstrate remarkable efficacy against ischemia-reperfusion injury due to their excellent biocompatibility, robust reactive oxygen species (ROS) scavenging capacity and anti-inflammatory properties. At the same time, urokinase (UK), a key pharmaceutical agent in antithrombotic therapy, has been effectively incorporated into the system, enhancing its ability to prevent and treat thrombosis. In addition, the integration of a platelet membrane (PM) has endowed the bionic vesicles with precise targeting and delivery capabilities, ensuring that the drugs can reach the lesion directly and facilitate efficient and precise release. The experimental results demonstrated that an ischemia-timed strategy can not only efficiently promote thrombolysis, but also effectively remove harmful elements in the microenvironment of ischemia-reperfusion injury. This discovery represents a new and promising approach to the treatment of thrombosis.

## Highlights

Novel bionic vesicles protect skin flaps via thrombolysis and reduce ischemia-reperfusion injury over ischemic durations.

• Bionic vesicle components have clinical histories, boosting translation to medical applications.
• Dual-target strategy addresses ischemia-reperfusion injury and thrombosis for better skin flap protection.
• Study links ischemia severity to ischemic time, guiding treatment timing and prevention strategies.
• Tailoring bionic vesicle properties enables personalized therapeutic strategies for patients.

## Graphical abstract

(a) Schematic diagram of the preparation of the bionic nanovesicles (UK-PBNZ@PM). (b) Mechanisms of the bionic nanovesicles (UK-PBNZ@PM) in microvascular cascade thrombolysis of skin flaps and prevention of ischemia-reperfusion injury. (UK: Urokinase; PBNZ: the Prussian blue nano-enzymes; PM: platelet membrane).

**Figure:**
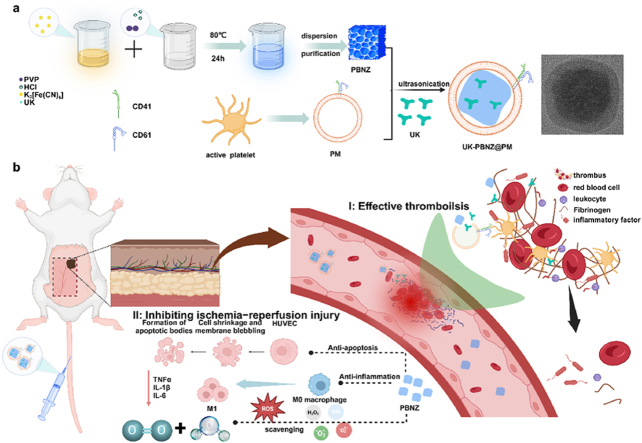

## 1 Introduction

Flap transplantation is currently the most dominant and effective method for the treatment of various tissue defects [1]. It has become the most effective method of restoring form and function to patients who have undergone resection of malignant tumors. Ischemia-reperfusion injury during skin flap transplantation is regarded as a pivotal and unavoidable factor that endangers the tissue and microvascular system, resulting in a considerable incidence of necrosis of the transplanted flap, ranging from 5 to 20 % [2,3]. The transfer of the flap from the donor to the recipient area inevitably entails periods of ischemia and subsequent reperfusion of blood supply, which can have a significant impact on flap's viability [4]. If the ischemic period exceeds the tolerance threshold of the tissue, the grafted flap is subjected to severe ischemia and hypoxia, depletion of ATP reserves, accumulation of harmful metabolites, and eventual destruction of cellular tissues, such as vascular endothelial cells. This process is exacerbated by the prolongation of the ischemic period [5]^.^ However, the adequate supply of oxygen and nutrients during the period of blood reperfusion paradoxically results in tissue damage through the generation of reactive oxygen species. Furthermore, it initiates a cascade of pathophysiological alterations, including endothelial cell dysfunction, diminished bioavailability of endogenous nitric oxide, positive feedback regulation that enhances ROS release, disturbances in energy metabolism, inflammatory cytokine release, and neutrophil aggregation, which collectively exacerbate the severity of flap injury. In addition, the use of flap grafts may cause vascular wall injury and torsion due to sutures [6]. This can lead to platelet adhesion and aggregation at the site of injury, as well as alterations in blood flow, which impede blood flow restoration [7]. A variety of methods have been employed to mitigate ischemia-reperfusion injury in grafted flaps, including pharmacologic therapy (such as antioxidants and free radical scavengers) [8,9], physical therapy (such as ischemic preconditioning and hypothermic preconditioning) [10,11], and surgical interventions (such as stem cell transplantation) [12]. Nevertheless, despite these endeavors, the clinically documented limitations of these methodologies persist, and they are predominantly “treating the symptoms” rather than addressing the underlying issue of ischemia-reperfusion injury. The pathophysiological mechanisms of flap ischemia-reperfusion injury are complex, with multiple mechanisms interacting with each other. This makes it challenging to achieve optimal results by targeting a single symptom. It is therefore imperative to develop new therapeutic methods and concepts to address the incidence of necrosis in transplanted flaps by improving the ischemia-reperfusion injury of the flap.

The prognosis of grafted flaps is significantly influenced by ischemia time, with the general consensus being that the prognosis of grafted flaps is worse with increasing ischemia time [13,14]. Nevertheless, the relationship between ischemia time and flap survival has not been extensively studied in clinical research. Some studies have even demonstrated that within 5 h, the reperfusion injury of lymphatic vessels improves with the prolongation of ischemia time. After 5 h, the degree of injury begins to correlate positively with the duration of ischemia [15]. At present, the majority of strategies employed to prevent grafted flap necrosis are limited to single thrombolytic therapy. This approach fails to acknowledge the significance of ischemia time as a pivotal variable, resulting in a lack of personalization and precision in treatment decision. In light of the considerable heterogeneity of circulatory disturbances among patients, a generalized treatment plan is frequently ineffective, resulting in an increased risk of bleeding due to over-treatment or a lack of thrombolysis due to under-treatment. It is therefore imperative to conduct a comprehensive investigation into the relationship between ischemia time and graft flap prognosis, with a view to developing a treatment strategy that can be flexibly adjusted according to the duration of ischemia. This would enable the precise thrombolysis and prevention of ischemia-reperfusion injury, thus optimizing the management of graft flaps and improving the therapeutic outcome. This represents a significant advancement in the existing treatment paradigm, as well as a crucial stride towards the realization of personalized and precision medicine.

Nano therapy combines the strengths of the chemical materials field and the biomedical field to provide a new strategy for the treatment and prevention of blood clots, with the starting point of personalized patient care and the aim of finding more accurate and efficient diagnostic and therapeutic techniques[[16], [17], [18]]. A plethora of nano therapies have been developed for thrombolytic therapy, including physically responsive nano-delivery systems (e.g., ultrasound, magnetically guided, shear force, etc.) [[19], [20], [21]] and biologically responsive nano-delivery systems (e.g., liposomes, biofilm-modified, and targeted peptide-modified nano-delivery systems)[[22], [23], [24]]. Nevertheless, the primary function of physically responsive nano-delivery systems is to disrupt the thrombus, although complete ablation is not achieved. In contrast, biofilm-modified nano-delivery systems have been demonstrated to exhibit superior performance, enabling precise targeting of the lesion and drug treatment. Furthermore, biofilm modification prolongs the half-life of the drug, mitigates the risk of bleeding associated with thrombolytic agents, and markedly enhances thrombolytic efficacy [25,26]. Recent studies have demonstrated that platelet membrane-modified nanodrug delivery systems can effectively target thrombus structures [27]. Urokinase has been demonstrated to exert a substantial inhibitory effect on early thrombosis and to possess high thrombolytic efficiency [28,29]. As an endogenous enzyme, urokinase is non-immunogenic and biologically safe, making it a promising thrombolytic therapy for a wide range of applications [30,31]. Functional combination of these three approaches has yet to be reported as a means of addressing the current difficulties in the treatment of flap ischemia-reperfusion injury and thrombosis.

It is also imperative to maintain vascular microenvironmental homeostasis in the treatment of ischemia-reperfusion injury. Ischemia-reperfusion injury frequently results in elevated levels of reactive oxygen species (ROS) within the flap, due to a number of underlying mechanisms [13,32,33]. This leads to oxidative stress within the flap as a whole and an inflammatory response, which is evidenced by increased secretion of inflammatory factors, including TNF-α, IL-1β, and IL-6, as well as the activation of inflammatory and apoptotic pathways within the tissue [[34], [35], [36]]. Nevertheless, the potential influence of microenvironmental homeostasis on the therapeutic outcome has not been sufficiently addressed in the existing therapeutic approaches for skin flap ischemia/reperfusion injury. This is a crucial area that requires immediate attention and investigation. Nanotherapeutics have demonstrated considerable potential in maintaining microenvironmental homeostasis, and their distinctive properties offer novel avenues for regulating and stabilizing the microenvironment. In vitro and in vivo studies have demonstrated that PBNZ exhibits notable anti-inflammatory, anti-apoptotic, anti-necrotic, and antioxidant activities in models of I/R-injured flaps. These findings suggest that PBNZ may play a crucial role in maintaining microenvironmental homeostasis during flap ischemia/reperfusion, which could contribute to the prevention and treatment of flap ischemia/reperfusion injury [37]. It is therefore imperative to introduce a personalized treatment concept based on an assessment of the ischemia time and prognosis. The objective of this concept is to achieve comprehensive and precise regulation of flap microenvironmental homeostasis through the integration of dual strategies: namely thrombolytic therapy and the prevention of ischemia-reperfusion injury. This therapeutic strategy holds both significant research value and promising clinical applications.

**Fig. 1:**
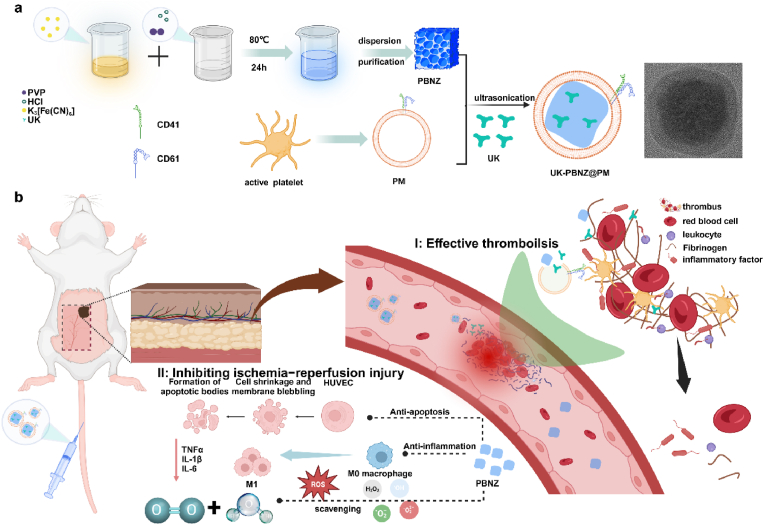
(a) Schematic diagram of the preparation of UK-PBNZ@PM. (b) Mechanisms of UK-PBNZ@PM in microvascular cascade thrombolysis of skin flaps and prevention of ischemia-reperfusion injury.

To address the complex challenge of thrombosis treatment and its accompanying ischemia-reperfusion injury, we have developed an innovative bionic vesicle nano-delivery system, UK-PBNZ@PM (Fig. 1a). This system deftly integrates the bionic properties of platelet membranes (PM), the thrombolytic potency of urokinase (UK), and the anti-inflammatory and antioxidant function of PBNZ, thereby achieving the dual objectives of thrombolysis and the prevention of ischemia-reperfusion injury. The UK-PBNZ@PM nano-delivery system was first successfully constructed by isolating pure platelet membranes from rat whole blood and efficiently loading PBNZ and UK into the membranes with the help of one-step ultrasound technology. The system exhibited remarkable efficacy at both the cellular and animal experimental levels. In vivo experiments unequivocally validated the remarkable capacity of the PBNZ element in mitigating inflammatory reactions and curbing oxidative stress (Fig. 1b). It effectively safeguarded the cells from oxidative damage induced by ischemia-reperfusion through the mechanisms scavenging reactive oxygen species (ROS) and inhibiting calcium inward flow. Moreover, the UK-PBNZ@PM nano-delivery system exhibited considerable protective effects and the potential for guiding medication administration in different varying ischemia times rat skin flap models. In comparison to the control group, the flaps in the drug-delivery group demonstrated superior recovery in both morphology and function, which suggests a promising potential for this system in the context of flap thrombosis treatment. In conclusion, the UK-PBNZ@PM nano-delivery system was designed to provide an innovative and effective solution for thrombolytic therapy and the prevention of ischemia-reperfusion injury, offering unique versatility, efficiency, and safety. This strategy not only enhances our comprehension of the utilization of nanotechnology in the biomedical field, but also inaugurates a novel approach to the management of skin flap thrombosis.

## 2 Materials and methods

### 2.1 Materials

Hydrochloric acid (HCl), polyvinylpyrrolidone (PVP, K30) and potassium ferricyanide (K_3_[Fe(CN)_6_]) were purchased from Sigma-Aldrich; Urokinase (Yuanye, China); CellROX™ Green Reagent (Thermo Fisher, USA); Peroxidase Assay Kit (Bioss, China); SOD Assay Kit (Dojindo Laboratories, Japan); Catalase Kit; Calcein-AM/PI Dual Staining Kit; JC-1 Enhanced Kit and Calcium Probe were purchased from Beyotime; Bovine Thrombin (Bersee, China); Fibrinogen (Solarbio, China); Annexin V-FITC/PI apoptosis detection kit was obtained from Vazyme (Nanjing, China). Mouse TNF-α, IL-1β, IL-6, Precoated ELISA Kit was purchased from Dakewei (Shenzhen, China).

Anti41(120–135 kDa) (PB9647) was purchased from Boster Technology; Integrin beta 3 Ab (CY5237); caspase-3 and cleaved capase-3 were purchased from Abway Technology; primary antibodies against P65 (T55034); p-P65 (TP56372); IκB-α (T55026) and p-IκB-α (TA2002) were purchased from Abmart Technology. Bax and Bcl-2 were purchased from Proteintech. A Tanon-5200 chemiluminescence imaging system (Tanon, China) was used to observe the membranes.

### 2.2 Preparation of PBNZ

PBNZ was synthesized according to the reported method with slight modifications [38]. PVP (8.0 g) and K_3_[Fe (CN)_6_] (696 mg) were mixed with HCl solution (1 M, 50 ml) and heated in a water bath at 80 °C for 1 h. The solution changed to a dark blue color, whereupon it was transferred to a vacuum drying oven and dried at 80 °C for 24 h. After drying, it was washed with deionized water five times and then centrifuged at 12,000 rpm for 20 min to obtain PBNZ.

### 2.3 Preparation of PM

The method of platelet membrane isolation was adapted from the literature [39], fresh blood was collected from the abdominal aorta of rats and placed in 4 % sodium citrate solution to prevent coagulation. Subsequently, the whole blood was centrifuged at 100g for 20 min to separate erythrocytes and platelets, resulting in platelet-rich plasma (PRP). the upper platelet rich plasma (PRP) was aspirated and PEG1 was added to inhibit platelet activation, and the step was repeated three times to enrich platelets. The platelet membrane was collected after centrifugation (800g, 20min) and finally resuspended with protease inhibitor (PMSF) and stored in a refrigerator at −80 °C.

### 2.4 Preparation of UK-PBNZ@PM

UK-PBNZ@PM was obtained by mixing 1 mg of UK and 1 mg of PBNZ in 1 ml of PBS containing platelet membranes and sonicating the mixture in an ice bath for 5 min, after which the solution was transferred to a 3500 Da dialysis bag and dialyzed slowly at 4 °C for 6 h and then lyophilized to obtain UK-PBNZ@PM. Similarly, ICG-PBNZ@PM and Ce6-PBNZ@PM were obtained by replacing UK with ICG and Ce6, respectively, in the aforementioned process.

### 2.5 Determination of drug encapsulation and embedding rate and drug release profile

1 mg of UK and 1 mg of PBNZ and 1 mg of platelet membrane were mixed in 1 ml of PBS and sonicated in an ice bath for 5 min. After sonication, the solution was dialyzed for 6 h and subsequently lyophilized. Following lyophilization, the drug release profiles were determined according to the following methods: Encapsulation Efficiency (EE%) = (mass of post-dialysis UK)/(mass of pre-dialysis UK) × 100 %, Drug Loading capacity (DL%) = (mass of post-dialysis UK)/(mass of pre-dialysis UK + PBNZ + PM) × 100 %. The UK-PBNZ@PM, which had been prepared successfully, was added to a 3000 Da dialysis bag and dialyzed in a 1 L beaker. The deionized water in the beaker was replaced every 6 h, and 100 μL of liquid in the dialysis bag was collected at 20-min intervals up to 12 h to determine the amount of un-released urokinase, calculate the release efficiency of urokinase, and plot the drug release curve.

### 2.6 Characterization and analysis of UK-PBNZ@PM

The hydrodynamic diameter of UK-PBNZ@PM nanoparticles was measured using a dynamic light scattering system (DLS, BT-90), and the morphology of PBNZ and UK-PBNZ@PM was observed using a scanning electron microscope (SEM, ZEISS GeminiSEM 300) and a transmission electron microscope (TEM, FEI Talos F200x). Ultraviolet–visible (UV–Vis) spectra of PBNZ were obtained using a UV–Vis–NIR spectrophotometer (PerkinElmer LAMBDA 750). The hydrodynamic diameter of PBNZ was measured using a dynamic light scattering system (DLS, BT-90). PBNZ and UK-PBNZ@PM were dissolved in DMEM (10%FBS) and PBS. The samples were stored at 4 °C for a period of seven days, with the dimensions monitored on a daily basis using DLS in order to evaluate their stability. FTIR (IRSpirit) was employed to ascertain whether the urokinase had been successfully loaded.

### 2.7 Characterization of platelet membrane integrity

The integrity of membrane proteins was characterized by sodium dodecyl sulphate-polyacrylamide gel electrophoresis (SDS-PAGE) of PBNZ, PM and UK-PBNZ@PM. Protein concentration was determined. The key proteins of the platelet membrane (CD41, CD61) were then determined according to routine Western blot experiments, while the integrity of the platelet membrane proteins on the bionic vesicle nano-delivery system (UK-PBNZ@PM) was verified by using Coomassie brilliant blue staining.

### 2.8 Assessment of CAT, POD and SOD enzyme activities

The respective enzyme activity capacities were determined using the Catalase (CAT) Activity Assay Kit, the POD Enzyme Kit and the SOD Enzyme Activity Kit according to the manufacturer's instructions.

### 2.9 Cytotoxicity

The cytotoxicity of UK-PBNZ@PM on human umbilical vein endothelial cells (HUVECs) and RAW264.7 cells was determined by the CCK-8 assay. HUVECs and RAW264.7 were inoculated into 96-well plates at a density of 1 × 10^4^ cells per well for 12 h and then incubated with different concentrations (0, 6.25, 12.5, 25, 50, 100, 200,400, 800 μg/ml) of UK-PBNZ@PM for 24 h. In the CCK-8 assay, cell viability (%) was reflected by absorbance at 450 nm. The live and dead cells were distinguished by Calcein-AM/PI Double Stain Kit.

### 2.10 Antioxidant damage resistance of PBNZ

HUVECs were cultured in six-well plates overnight to await cell attachment. Cells were pretreated with different concentrations of PBNZ for 12 h, followed by treatment with 600 μM hydrogen peroxide for 4 h. Untreated cells were used as control. Cell viability (%) was measured by absorbance at 450 nm according to the CCK-8 method; ROS content was detected by staining with DCFH-DA (5 μM) for 30 min, followed by PBS rinsing, and then the cells were imaged using an inverted fluorescence microscope and the average fluorescence intensity was quantified using ImageJ.

### 2.11 Anti-inflammatory capacity of PBNZ

RAW264.7 was cultured in six-well plates overnight to allow the cells to attach to the walls. The cells were then divided into two groups, one pre-treated with PBNZ (100 μg/ml) for 12 h and one untreated, after which one dish from each group was stimulated with 1 μg/ml LPS at 37 °C for 24 h. The rest of the cells were cultured normally without treatment and the cells harvested. The expression of intracellular associated proteins was analyzed by Western blot, and the supernatant from the cell culture medium was collected and centrifuged at 12,000 rpm for 10 min. The expression of inflammatory factors in the supernatant was detected by ELISA (Dakewei, China) according to the manufacturer's protocol.

### 2.12 Oxygen and glucose deprivation model

HUVECs (3 × 10^4^) were cultured in six-well plates and adhered overnight. After two days of culture, until the cell confluence reached 80 %, they were starved with low-glucose DMEM (1.0 g/ml) medium containing UK-PBNZ@PM (0–200 μg/ml) for 12 h [40]. Cells of the H/R group were cultured in a wet hypoxia incubator (PH-1-A) under no-sugar, no-FBS, low oxygen (1 %, O_2_) conditions (PH-1-A, Puhebio, Wuxi, China). After 3h, 6h or 9h of hypoxia, the cells were re-exposed to normal O_2_ conditions (95 % air and 5 % CO_2_) using standard medium. After 1 h, the cells were harvested for further analysis. The control group was cultured in normal medium under normal O_2_ conditions. HUVECs treated with I/R (3/1h, 6/1h, 9/1 h) were harvested, and changes in cell viability after different treatments were determined by CCK-8, apoptosis was detected by Annexin V-FITC/PI apoptosis detection kit, and changes in expression of apoptosis pathway-related proteins were detected by Western blot, respectively.

### 2.13 Changes in mitochondrial function in ischemia-reperfusion injury

According to the construction of the above oxygen and glucose deprivation model, HUVECs were co-cultured with UK-PBNZ@PM (100 μg/ml), and the changes in mitochondrial membrane potential (Δψm) were detected using the JC-1 probe for the 3h, 6h, and 9h groups, respectively. Cells were imaged by confocal laser scanning microscopy (TCS-SP5, Leica) and quantified by flow cytometry. Red and green fluorescence corresponded to JC-1 aggregates and monomers, respectively, and Δψm corresponded to the ratio of red and green fluorescence intensities; intra- and extracellular calcium ion concentrations were quantified following the instructions for use of the Calcium Ion Kit.

### 2.14 Validation of in vitro thrombogenicity

We added 200 μL fibrinogen, 20 μL CaCl_2_ (0.2 mol/L), 2 μL thrombin (100 U/ml) and FITC dye to a confocal petri dish and incubated for 3 h at 37 °C in the absence of light to form a FITC-linked fibrin clot. Ce6-PBNZ, Ce6-PBNZ@PM and PM + Ce6-PBNZ@PM were co-cultured for 20 min and observed by confocal microscopy.

### 2.15 Validation of in vitro thrombolytic capacity

Evaluation of in vitro thrombolytic activity Thrombolytic activity was evaluated by the ratio of weight loss after treatment. First, the artificial thrombus from SD rats was cut into equal pieces and weighed, labeled as the original weight. The thrombus was placed in a 6-well plate for static thrombolysis and randomly divided into 3 groups (n = 3 in each group): PBS, UK, and UK-PBNZ@PM at a concentration of 100 mg/ml. The treatment time was set to 90min. After the treatment, filter paper was used to remove surface water, the thrombus pieces were reweighed, and the weights were marked as the final weights. Finally, the thrombolytic rate was calculated. The thrombolytic activity was determined as follows: thrombolytic rate (thrombolytic activity) = (initial weight - final weight)/(initial weight) × 100 %.

### 2.16 Animal skin flap ischemia-reperfusion model

SD rats (6-week-old males, 200–300g) were purchased from Vital River Animal Laboratory and housed in an SPF grade animal room with a light-dark cycle at 22 °C for 12 h. Food and water were provided ad libitum. The experimental animal protocol was approved by the Institutional Animal Care and Use Committee (IACUC) of Nanjing University School of Medicine.SD rats (6w, males) were anaesthetized with chlorofluorocarbon gas. The surgical area was prepared with hair, sterilized with iodophor and covered with sterile gauze. An axial skin flap of 3 cm × 6 cm was drawn in the abdomen of the rat with the rotating anterior artery of the abdominal wall as the tip, and the flap was removed with microsurgical instruments and then the vessel tip was clamped with a microvascular clip to completely block the blood flow for 3h, 6h, or 9h. The clip was removed and the flap was sutured to its original position with 4-0 stitches. A rat skin flap I/R injury model was thus established. The rats were randomly divided into twelve groups (n = 3), namely sham-operated group, sham-operated group + UK-PBNZ@PM, sham-operated group + UK, 3h group, 3h + UK-PBNZ@PM, 3h + UK, 6h group, 6h + UK-PBNZ@PM, 6h + UK, 9h group, 9h + UK-PBNZ@PM group, 9h + UK. The UK group and UK-PBNZ@PM group were given medication once a day 1 h before surgery and in the first 3 days after surgery. Seven days later, the flap tissue was removed and photographed, and the necrotic area was calculated using ImageJ software. They were then fixed in paraformaldehyde. All flaps were 1 cm × 1 cm in size and the distal vascular end of the flap was in the same position. H&E staining was used to detect inflammatory cell infiltration in the tissue, and immunohistochemical staining was used to detect the expression of inflammatory substances (TNF-α, IL-6, IL-1β) and apoptosis-related proteins (Bax, Bcl-2) in the flap.

### 2.17 Animal skin flap vessel tip thrombosis model

The male rats, aged six weeks, were anaesthetized with isoflurane. The surgical area was prepared, disinfected with iodophor, and covered with sterile gauze. A 3 cm × 6 cm axial skin flap was created on the abdomen of the rat, with the abdominal wall and the anterior superior iliac vein serving as the pedicle. A 5 mm × 5 mm filter paper soaked in a 10 % ferric chloride solution was used to wrap the right abdominal wall anterior superior iliac vein of the rat for a period of 10 min, thereby creating a model of flap thrombosis.

### 2.18 In vivo thrombolytic effect assay

The rats were randomly divided into three groups. After the thrombus formation model was established, ICG drug was injected into the rats to develop and thrombus formation was observed, then PBS, UK and UK-PBNZ@PM were injected respectively, and the ICG development was observed under the NIR-II in vivo imaging system after 2h to judge the degree of vascular recanalization; The rats were then euthanized and the tibial vascular tissues were harvested, and the embedded sections were fixed in paraformaldehyde and then stained with HE stain, and the different degrees of thrombolysis were quantified and analyzed.

### 2.19 Biocompatibility of drugs in vivo

Rats were randomly divided into UK-PBNZ@PM (1 mg/kg, 100 μL, administered via tail vein) and a control groups, and major organs were stained with HE stains, and blood was collected from the abdominal aorta after 7 days. The effect of drug dose on coagulation time and biochemical indices was studied in vivo.

### 2.20 Data analysis

Data were analyzed using Origin 2023, FlowJo, ImageJ and GraphPad 8 software. All error bars are expressed as mean ± SD. Statistical analyses were performed by one-way analysis of variance (ANOVA) using GraphPad Prism 8.01. Differences were considered statistically significant (∗P < 0.05, ∗∗P < 0.01, ∗∗∗P < 0.001 and ∗∗∗∗P < 0.0001).

## 3 Results

### 3.1 Preparation and characterization of UK-PBNZ@PM

**Fig. 2:**
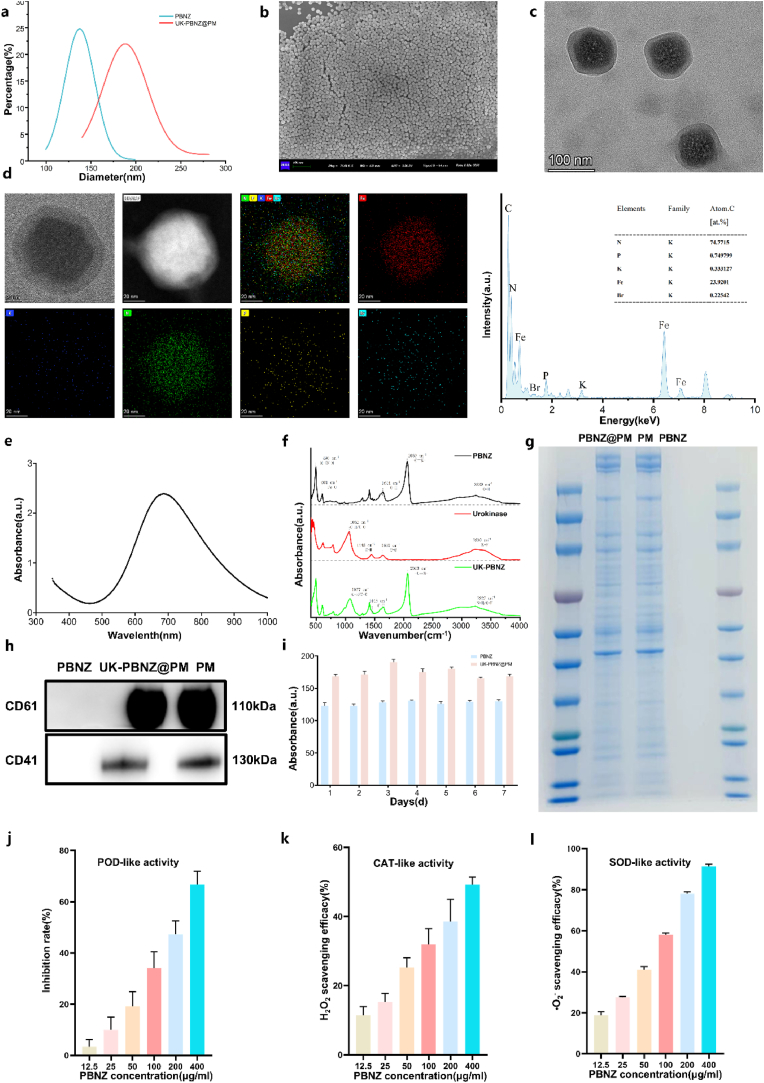
Construction, characterization and reactive oxygen species scavenging performance of UK-PBNZ@PM biomimetic vesicles. (a) The mean size of PBNZ and UK-PBNZ@PM. (b) Scanning electron microscopy (SEM) image of PBNZ. Scale bar = 200 nm (c) Scanning transmission electron microscopy (STEM) image of UK-PBNZ@PM. (d) The STEM image of UK-PBNZ@PM, along with corresponding STEM-energy dispersive spectrometer (STEM-EDS) elemental mapping and line scanning showing the distribution of Fe, K, N, P and Br. Scale bar = 20 nm. (e) Ultraviolet-visible-near infrared (UV-vis-NIR) absorbance spectra of PBNZ. (f) FTIR spectra of PBNZ, UK and UK-PBNZ. (g) Coomassie blue staining of PBNZ, PM and PBNZ@PM. (h) CD41 and CD61 expression in PBNZ, UK-PBNZ@PM and PM. (i) Change in particle size of PBNZ and UK-PBNZ@PM in DMEM over one week. (j) Peroxidase activity (n = 3). (k) H_2_O_2_ scavenging efficiency (n = 3). (l) •O_2_^−^scavenging efficiency (n = 3). (For interpretation of the references to color in this figure legend, the reader is referred to the Web version of this article.)

To achieve a dual strategy of thrombolytic therapy and prevention of ischemia-reperfusion injury, we have developed UK-PBNZ@PM, in which platelet membranes (PM) have been modified onto PBNZ particles loaded with urokinase (UK). This system ingeniously integrates the biomimetic properties of PM, the thrombolytic efficacy of UK, and the anti-inflammatory and antioxidant functions of PBNZ. The detailed physicochemical characterization results indicate that UK-PBNZ@PM possesses excellent potential for biomedical applications. In particular, the results of the dynamic light scattering (DLS) analysis demonstrated that the particle sizes of PBNZ and UK-PBNZ@PM were 137.51 ± 1.05 nm and 188.03 ± 2.53 nm, respectively (Fig. 2a). Scanning electron microscopy (SEM) images revealed that PBNZ exhibited a cubic morphology with good dispersion and uniform particle size. Following the coating of UK-PBNZ with PM, a distinctive core-shell structure was observed, consisting of a cubic PBNZ core encased in a platelet membrane shell (12–18 nm thick), exhibiting good dispersion (Fig. 2b and c). Elemental mapping confirmed the presence of phosphorus (P) and bromine (Br) on the surface of UK-PBNZ@PM, thereby verifying the successful incorporation of platelet membranes and urokinase onto the nanoparticle surface (Fig. 2d). The absorption spectrum of PBNZ shows a distinct peak near 700 nm, which can be attributed to the electronic transition of the Fe-CN-Fe group in Prussian blue (Fig. 2e) [41]. The results of the Fourier Transform Infrared Spectroscopy (FT-IR) analysis, featuring prominent peaks at 2063 cm-1, 3227 cm-1, 1077 cm-1, and 1637 cm-1, provide evidence that the synthesis of Prussian Blue and its conjugation with urokinase were successful (Fig. 2f). The BCA Protein Assay Kit was employed to determine the loading efficiency of UK onto the carrier, which was found to be 73.39 % ± 3.18 %. Subsequently, based on the standard concentration calibration curve of PBNZ (as illustrated in Figures S2 and S4), the drug loading capacity of the carrier was calculated with precision to be 26.84 % ± 0.76 %. Moreover, the drug release profile over time from the carrier has been plotted in Figure S3, which provides a visual representation of the dynamic changes in release behavior. Subsequently, SDS-PAGE analysis of the UK-PBNZ-PM complex, stained with Coomassie Blue, revealed a protein pattern highly like that of platelets (Fig. 2g), indicating the successful preservation of key structural features. Moreover, western blot analysis demonstrated that UK-PBN@PM retained crucial membrane adhesion proteins CD41 and CD61 (Fig. 2h), thereby validating its functional mimicry and establishing a robust foundation for biomedical applications.

Moreover, a preliminary assessment of the storage stability of UK-PBNZ@PM was conducted. In a serum-containing medium, UK-PBNZ@PM exhibited excellent colloidal stability, indicating its capacity to maintain a stable state within the blood environment (as illustrated in Fig. 2i). This finding provides substantial evidence in support of its potential applications in the biomedical field. Among the numerous enzymatic activities of PBNZ, we focused on validating its peroxidase-like activity. This distinctive enzymatic activity enables PBNZ to catalyze the decomposition of hydrogen peroxide, thereby promoting the oxidation of phenolic and amine compounds. This characteristic not only effectively eliminates the harmful effects of hydrogen peroxide but also mitigates the toxicity of phenolic and amine substances, thereby achieving a dual detoxification effect. As illustrated in Fig. 2j, PBNZ displays notable peroxidase (POD) mimicry activity, which is of considerable significance in the domain of biomimetics. In addition to this, PBNZ has the capacity to mimic the function of catalase (CAT), efficiently catalyzing the decomposition of hydrogen peroxide (H₂O₂) into water (H₂O) and oxygen (O₂), thereby effectively preventing the excessive accumulation of H₂O₂ in biological systems and protecting cells and tissues from oxidative damage caused by it. Further analysis of the data presented in Fig. 2k revealed a positive correlation between the decomposition efficiency of hydrogen peroxide (H₂O₂) and the concentration of PBNZ. It is noteworthy that at a concentration of 400 μg/ml, the decomposition rate of hydrogen peroxide increased significantly to 49.20 %. This confirms the outstanding performance of PBNZ as a catalase mimic in scavenging hydrogen peroxide.

Superoxide dismutase (SOD) is a vital class of enzymes that strengthens a robust antioxidant defense within cells by catalyzing the dismutation of superoxide anions (•O_2_^−^) into oxygen (O_2_) and hydrogen peroxide (H_2_O_2_). The present study observed a significant increasing trend in the percentage of •O_2_^−^ inhibition with the gradual increase of PBNZ concentration (as demonstrated in Fig. 2l), which is highly consistent with the highly efficient SOD-like activity demonstrated by the PBNZ. This further confirms their potential in mimicking the function of natural antioxidant enzymes. To gain a more comprehensive understanding of the efficacy of PBNZ in scavenging reactive oxygen species (ROS), we conducted a series of more detailed experiments. In particular, the introduction of PBNZ into a hydrogen peroxide solution (7.5 %) in a cuvette resulted in the formation of numerous bubbles within a relatively short period of time. In contrast, the control sample of pure hydrogen peroxide solution did not exhibit any significant changes (as illustrated in Figure S5). This phenomenon provides compelling evidence of the exceptional ROS scavenging ability of PBNZ, which is capable of rapidly and effectively neutralizing harmful hydrogen peroxide molecules. Considering the above experimental results, it seems reasonable to posit that PBNZ, with its exceptional ROS scavenging properties, may play a pivotal role in high-ROS microenvironments during pathophysiological processes such as ischemia-reperfusion. It has the potential to provide effective protection to cells, mitigating or reversing ROS-mediated tissue damage, thereby demonstrating a broad range of potential clinical applications.

### 3.2 The anti-inflammatory and antioxidant damage-reducing capacity of PBNZ has been demonstrated

Following ischemia-reperfusion injury in skin flaps, a substantial oxidative stress microenvironment is formed, distinguished by the surge of oxygen-derived free radicals, mitochondrial dysfunction, elevated oxidation of catecholamines, and augmented metabolism of arachidonic acid. These phenomena collectively result in cell membrane damage, protein denaturation, and disruption of energy metabolism. Furthermore, the interplay between oxidative stress and the inflammatory response creates a vicious cycle that further exacerbates tissue damage. Consequently, a comprehensive approach incorporating antioxidant, anti-inflammatory, microcirculation-improving, and cell-protective strategies is required to address this pathological process, with the aim of mitigating tissue injury and facilitating repair.

**Fig. 3:**
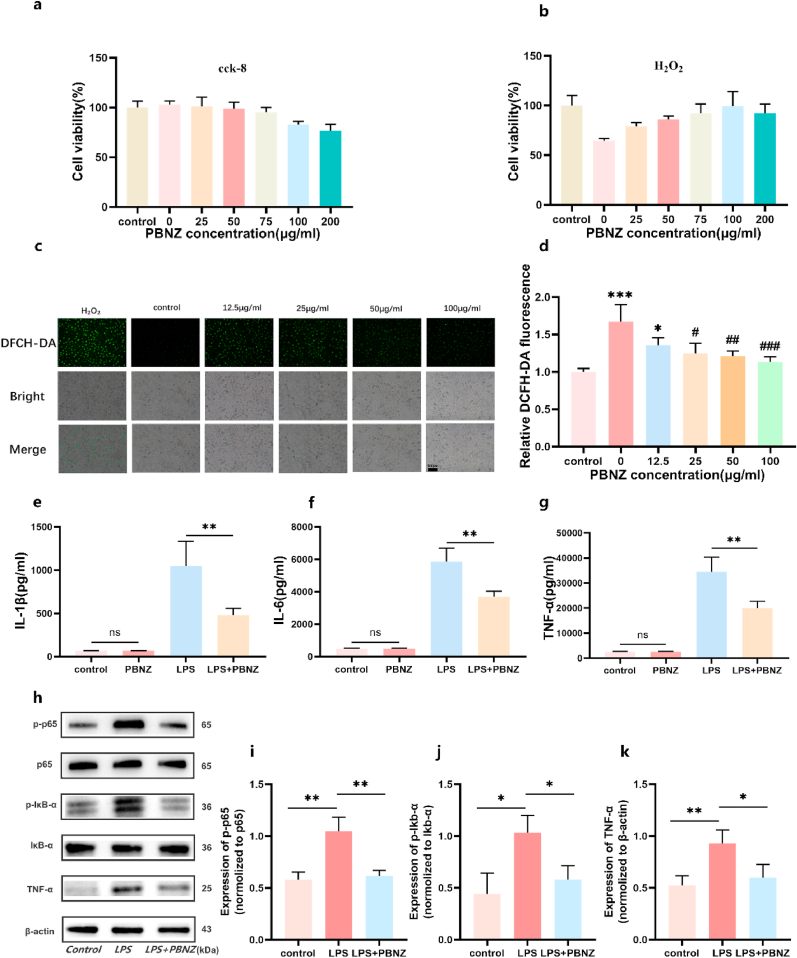
In vitro antioxidant damage and anti-inflammatory activity of PBNZ. (a) Cytotoxicity of PBNZ on HUVECs. (b) Protective effect of PBNZ on HUVECs treated with H_2_O_2_. (c) Fluorescence microscopy detects reduced ROS levels in the HUVECs model stimulated with H_2_O_2_ after PBNZ treatment. Scale bar: 100 μm. (d) Quantification of fluorescence in (c). (n = 3; ∗compared with control group: ∗p < 0.05; ∗∗∗: p < 0.001; # compared to the positive control group: #: p < 0.05; ##: p < 0.01; ##: p < 0.001). (e–g) RAW264.7 cells stimulated with LPS for 24 h with or without PBNZ; ELISA to detect IL-1β, IL-6 and TNF-α levels. (h) Western blot images of p65, p-p65, IκB-α, p-IκB-α and TNF-α protein expression Western blot images. (i–k) p-IκB-α/IκB-α, p-p65/p65 and TNF-α/β-actin protein relative expression levels (n = 3; ∗: p < 0.05; ∗∗: p < 0.01; ns: no significant difference).

To investigate the antioxidant and anti-inflammatory properties of the novel material, PBNZ, in the context of ischemia-reperfusion injury. An in vitro glucose-oxygen deprivation model was established using HUVEC and mouse macrophages (RAW264.7). The primary objective was to evaluate the capacity of PBNZ to scavenge reactive oxygen species (ROS) and mitigate inflammatory responses in this simulated environment. Initially, we ensured the validity and safety of our experiments by assessing the toxicity of PBNZ towards the target cells using the CCK-8 cell proliferation assay and Calcein-AM/PI double staining. The results demonstrated that PBNZ displayed excellent biocompatibility with both cell types, maintaining high cell viability up to a concentration of 200 μg/ml (as illustrated in Fig. 3a–S6, and S7), thus establishing a robust foundation for subsequent functional studies. Subsequently, an oxidative stress model was constructed by exposing HUVECs to H₂O₂, thereby mimicking the pathological state that occurs following ischemia-reperfusion. In this model, cells treated solely with H₂O₂ exhibited a significant reduction in cell viability, reaching a value of 64.62 ± 2.2 %. However, pretreatment with PBNZ was observed to confer a protective effect, with cell viability gradually increasing as the concentration of PBNZ rose, reaching a peak of 99.40 ± 14.6 % at 100 μg/ml (Fig. 3b). Of note, a slight decline in cell viability (92.27 ± 9.2 %) was observed at 200 μg/ml, likely due to mild toxicity associated with the higher concentration. To substantiate the assertion that PBNZ's cytoprotective effects are attributable to its ROS-scavenging capabilities, we employed the DCFH-DA fluorescent probe to quantify intracellular ROS levels. In comparison to the control cells that had not been subjected to any treatment, the cells that had been exposed to H₂O₂ for a period of 4 h exhibited a considerable elevation in ROS levels. In contrast, pretreatment with 100 μg/ml PBNZ resulted in a significant reduction in ROS levels, which were restored to levels like those observed in the control group. This finding corroborates the dose-dependent ROS-scavenging activity of PBNZ (as illustrated in Fig. 3c and d). Based on these observations, we hypothesize that PBNZ, through its catalase (CAT)-mimicking activity, efficiently decomposes H₂O₂ into harmless oxygen and water, thereby mitigating its cytotoxic effects and effectively safeguarding against oxidative damage. Later, we put it into the glucose-oxygen deprivation model for verification and found that the ROS concentration decreased significantly after PBNZ treatment, proving that PBNZ has an excellent protective effect in the glucose-oxygen deprivation model (as illustrated in Figures S9). In conclusion, our study not only corroborates the safety of PBNZ in vitro towards HUVECs and RAW264.7 cells but also highlights its remarkable potential in eliminating ROS and protecting cells from oxidative stress. These findings present novel insights and promising candidates for therapeutic strategies aimed at addressing ischemia-reperfusion injury.

In examining the complex mechanisms of ischemia-reperfusion injury (IRI), we observed that various endogenous molecules activate specific signaling pathways, including the Toll-like receptor (TLR)-mediated downstream cascade, which in turn activates nuclear factor-κB (NF-κB). This subsequently induces the release of proinflammatory cytokines, such as Tumor necrosis factor-α (TNF-α), interleukin-1β (IL-1β), and interleukin-6 (IL-6) proinflammatory cytokines and chemokines that are released in response to the above mechanism. This process serves to exacerbate the infiltration of inflammatory cells into the affected tissues [42]. To simulate this inflammatory response, we constructed an in vitro cellular inflammatory model by stimulating RAW264.7 macrophages with lipopolysaccharide (LPS). In the experimental design, 100 μg/ml of PBNZ was introduced as a pretreatment for a period of 12 h. Subsequently, the concentration changes of TNF-α, IL-1β, and IL-6 in the cell culture supernatants were quantified, revealing that PBNZ significantly inhibited the release of these crucial proinflammatory cytokines (Fig. 3e–g). This initial observation revealed the anti-inflammatory potential of PBNZ. To gain further insight into the anti-inflammatory mechanism of PBNZ, we focused on the classical NF-κB inflammatory signaling pathway. Western blot analysis was employed to systematically evaluate the protein expression levels of TNF-α, IκB-α (the inhibitor protein of NF-κB), its phosphorylated form p-IκB-α, as well as the nuclear factor NF-κB p65 and its phosphorylated form p-p65 (Fig. 3h–k). The results demonstrated that, in comparison to cells stimulated with LPS alone, the protein expressions of TNF-α, p-IκB-α, and p-p65 were markedly diminished in the PBNZ-pretreated group. Considering these findings, it seems reasonable to posit that PBNZ inhibits the phosphorylation of IκB-α, thereby blocking the nuclear translocation of NF-κB p65 and its subsequent transcriptional activity. This, in turn, leads to the downregulation of NF-κB-dependent inflammatory gene expression such as TNF-α. This process represents the fundamental molecular mechanism underlying the anti-inflammatory effects of PBNZ. In conclusion, this study not only validates the anti-inflammatory efficacy of PBNZ in an in vitro model but also provides preliminary insights into its specific mechanism of inhibiting inflammatory responses through modulation of the NF-κB signaling pathway. These findings offer a theoretical foundation and experimental evidence for the potential application of PBNZ and similar compounds in the treatment of inflammatory diseases.

### 3.3 Protective role of PBNZ in mitigating cellular damage during ischemia-reperfusion injury via mitochondrial preservation

**Fig. 4:**
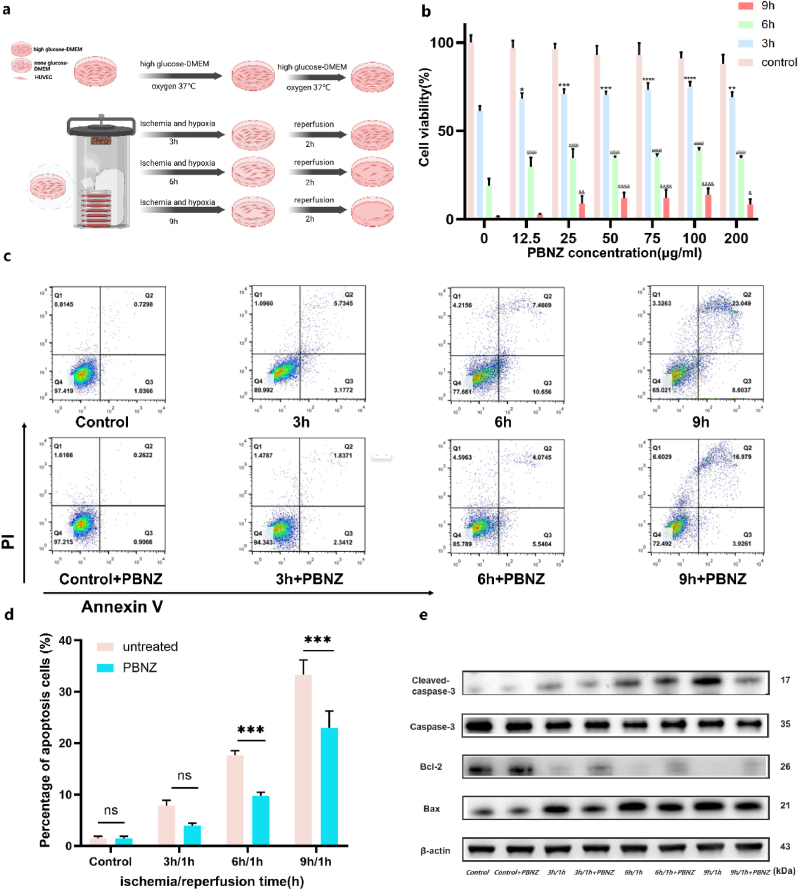
Protective effect of PBNZ on HUVECs model of hypoxia-reperfusion injury. (a) Model diagram of ischemia-reperfusion injury model. (b) CCK-8 assay of the protective effect of PBNZ (μg/ml) on HUVECs with different ischemic times. (n = 3; ∗, #, &: p < 0.05; ∗∗, ##, &&: p < 0.01; ∗∗∗, ###, &&&: p < 0.001; ∗∗∗∗, ####, &&&&: p < 0.0001; ns: no significant difference). (c) Flow cytometry analysis showed that PBNZ (100 μg/ml) had a protective effect on HUVECs under different ischemic time (3h, 6h, and 9h) treatments. Images show representative results from one of three independent experiments. (d) Quantitative analysis of C-maps (n = 3; ∗∗∗: p < 0.001; ns: not significantly different). (e) Western blot images of cleaved caspase-3, cleaved-caspase-3, Bax and Bcl-2 protein expression in HUVECs after PBNZ pretreatment under different H/R (3/1 h, 6/1h, and 9/1h) conditions.

Considering PBNZ's noteworthy anti-inflammatory and anti-oxidative stress properties, we undertook a comprehensive investigation into its protective effects against cellular ischemia-reperfusion injury. An in vitro glucose-oxygen deprivation model was constructed using HUVECs as the model system, with the objective of mimicking the physiological process of ischemia followed by reperfusion. In particular, the cells were subjected to hypoxia (1 % oxygen) in conjunction with glucose- and serum-free conditions, which were used to simulate ischemia. This was followed by exposure to normal oxygen levels, high-glucose media, and 10 % serum, which were used to mimic reperfusion (Fig. 4a). Initially, the CCK-8 assay was employed to assess cell viability after various durations of ischemia (3h, 6h, 9h) followed by 1 h of reperfusion. Our findings revealed a significant reduction in cell viability with prolonged ischemia. Cell viability initially reached 100 ± 3.7 %, but declined to 61.28 ± 2.58 % after 3 h, 19 ± 3.72 % after 6 h, and to near-negligible levels of 1.39 ± 0.25 % after 9 h. These results highlight the considerable impact of cumulative ischemia on cellular survival. To validate the protective efficacy of PBNZ, HUVECs were pretreated with varying concentrations of PBNZ for 12 h prior to ischemia-reperfusion conditions. It is noteworthy that PBNZ pretreatment significantly enhanced cell viability, with the optimal concentration of 100 μg/ml eliciting peak cell viability across all ischemia durations (75.06 ± 2.44 %, 38.83 ± 1.5 %, and 13.96 ± 3.2 %, respectively). However, at a higher concentration of 200 μg/ml, cell viability declined, indicating the potential onset of cellular toxicity and a subsequent compromise in protective efficacy (Fig. 4b). Subsequently, 100 μg/ml PBNZ was identified as the optimal pretreatment concentration, and the Annexin V-FITC/PI apoptosis detection kit was employed to assess the capacity of PBNZ to inhibit apoptosis. The results demonstrated a significant increase in apoptosis rates with prolonged ischemia, reaching a peak of 33.32 ± 2.34 % after 9 h in untreated cells. In contrast, PBNZ pretreatment markedly reduced apoptosis to 22.99 ± 2.65 % (Fig. 4c and d), confirming its efficacy in mitigating ischemia-reperfusion-induced apoptosis. Finally, to elucidate the underlying molecular mechanisms of PBNZ's protective effects, Western blot analysis was conducted to assess the expression of key apoptotic regulatory proteins. In cells that had not been treated with PBNZ, a time-dependent increase in the levels of two pro-apoptotic proteins, cleaved caspase-3 and Bax, was observed, accompanied by a decrease in the levels of an anti-apoptotic protein, Bcl-2. These changes are indicative of the activation of apoptotic signaling pathways. In contrast, PBNZ pretreatment reversed these trends, with reduced cleaved-caspase-3 and Bax expression and increased Bcl-2 expression. This provides direct evidence that PBNZ exerts its protective effects by modulating the expression of apoptosis-related proteins to inhibit apoptosis and safeguard cells against ischemia-reperfusion injury (Fig. 4e). This study not only highlights the potential of PBNZ in cellular protection but also provides valuable insights for the development of novel therapeutics against ischemia-reperfusion injury.

**Fig. 5:**
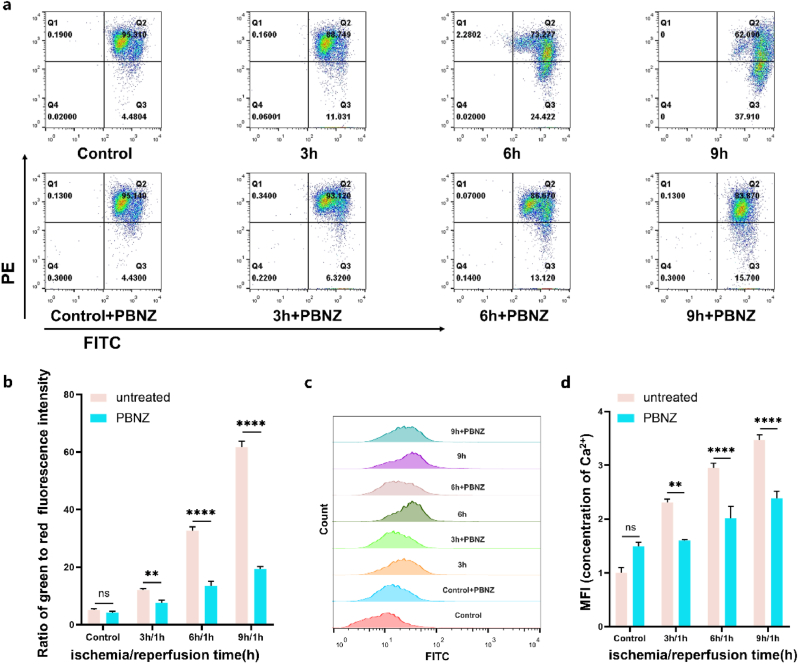
Protective effect of PBNZ enzyme on mitochondria in ischemia-reperfusion injury. (a) Measurement of mitochondrial membrane potential (Δψm) level in HUVECs with JC-1 probe. (b) Quantitative analysis of JC-1 flow-through results. (n = 3; ∗∗: p < 0.01; ∗∗∗∗: p < 0.0001; ns: no significant difference). (c) Mitochondrial calcium levels in HUVECs were measured using the Fluo-4 AM probe. Images show representative results from one of three independent experiments. (d) Quantitative analysis of calcium ion concentration based on the flow chart results (n = 3; ∗∗∗∗: p < 0.0001; ns: no significant difference).

In examining the mechanisms underlying the protective role of PBNZ in mitochondria during ischemia-reperfusion injury, we concentrated on the pivotal regulatory elements of mitochondrial apoptosis, with a particular emphasis on the stability of the mitochondrial membrane potential (Δψm) and the maintenance of mitochondrial calcium ion (Ca^2^⁺) homeostasis [43]. Mitochondria, as the central regulators of cellular apoptosis, are subject to intricate regulation by the delicate balance of pro-apoptotic and anti-apoptotic proteins within the Bcl-2 family. In the context of ischemia-reperfusion, mitochondria are confronted with a multitude of stressors, including the production of excessive reactive oxygen species (ROS), DNA damage, disruption of calcium ion homeostasis, and membrane depolarization [44]. These factors serve as pivotal triggers of cellular apoptosis. To evaluate the influence of PBNZ on mitochondrial membrane potential, we utilized the highly sensitive JC-1 probe. The experimental results demonstrated that, in comparison to the control group, HUVECs that underwent ischemia-reperfusion for varying durations (3h, 6h, 9h) exhibited a gradual decline in mitochondrial membrane potential, indicating a progressive deterioration of mitochondrial function. However, when cells were pretreated with PBNZ prior to ischemia, the decline in mitochondrial membrane potential was significantly attenuated at equivalent ischemia durations, demonstrating that PBNZ effectively inhibits mitochondrial membrane depolarization and thereby maintains mitochondrial functional stability (Fig. 5a and b). Moreover, changes in mitochondrial calcium ion concentrations were monitored using the Fluo-4 AM probe. As the duration of ischemia increased, the levels of calcium ions within the mitochondria of HUVECs rose significantly, as evidenced by flow cytometry analysis. This phenomenon is a hallmark of mitochondrial dysfunction and a precursor to cellular apoptosis. It is noteworthy that PBNZ pretreatment effectively reversed this trend, thereby aiding the cells in maintaining mitochondrial calcium ion homeostasis (Fig. 5c and d). Considering these findings, it can be concluded that the reduction in mitochondrial membrane potential and the increase in calcium influx both indicate abnormal mitochondrial function, which subsequently triggers intracellular apoptotic pathways, ultimately leading to cell death. The pretreatment effect of PBNZ, which mitigates the extent of mitochondrial dysfunction and reduces the expression of apoptosis-related proteins, effectively prevents and protects cells from oxidative damage. This discovery not only deepens our understanding of PBNZ's protective mechanisms but also provides robust support for the development of novel therapeutic agents targeting mitochondrial injury.

### 3.4 UK-PBNZ@PM possesses precise thrombus targeting capabilities and superior thrombolytic performance

**Fig. 6:**
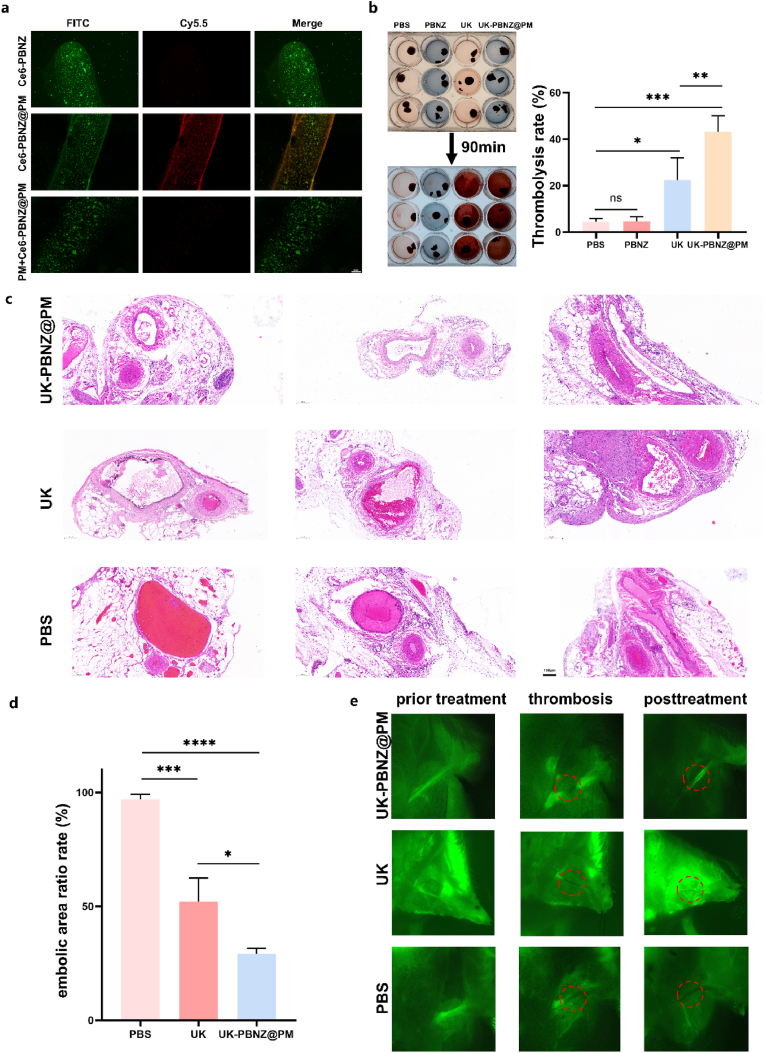
Targeting and thrombolytic ability of UK-PBNZ@PM. (a) In vitro binding ability of nano-enzymes to fibrin-FITC under confocal microscopy; FITC signaling image of fibrin coagulation (left), Cy5.5 signaling image of Ce6-PBNZ@PM binding (middle) and merge image (right). (b) Images and quantitative analysis of PBS, PBNZ, UK (100U/g) and UK-PBNZ@PM (100U/g) after co-incubation with in vitro blood clot for 90 min. (c) HE staining of abdominal flap thrombus. (d) Embolism rate analyzed by Image J (n = 3; ∗: p < 0.05; ∗∗∗: p < 0.001; ∗∗∗∗: p < 0.0001; ns: no significant difference). (e) ICG imaging of abdominal vessels before abdominal flap (left), ICG imaging of abdominal vessels after thrombus formation (middle) and abdominal vessels after UK-PBNZ@PM, UK and PBS treatment (right).

Biofilm-modified nanodrug delivery systems have emerged as a prominent area of research in the biomedical field, distinguished by their unique advantages. These systems have the capacity to target disease sites with precision, minimize drug distribution in non-target areas, enhance therapeutic efficacy, and reduce adverse effects. Among the various membrane-based nanocarriers, platelet membrane nanocarriers are particularly effective in the treatment of thrombotic disorders due to their immune evasion capabilities, high biocompatibility, robust drug protection and controlled release mechanisms, as well as their multifunctionality. In our pursuit of an efficacious targeted thrombolytic strategy, we concentrated our efforts on fibrinogen, the primary component of thrombi, and corroborated the specific binding capacity of activated platelet membranes (PM) towards it. To visualize the interactions between bovine fibrinogen and PBNZ, a labeling system was established whereby fibrinogen was labeled with fluorescein isothiocyanate (FITC) and PBNZ was labeled with Chlorin e6 (Ce6). The experimental results demonstrated that the Ce6-PBNZ@PM group exhibited intense red fluorescent signals, which were in stark contrast to the green fluorescent signals of fibrinogen. In comparison, the other control groups exhibited weaker red fluorescence. This finding robustly confirmed the high affinity of activated platelet membranes for fibrinogen, thereby establishing the foundation for the development of biomimetic vesicular nanodrug delivery systems with precise targeting capabilities (Fig. 6a). To further validate the in vitro targeting efficiency of this system, a fresh thrombus model was prepared and the behavior of indocyanine green (ICG)-loaded biomimetic vesicular nanodrug delivery systems was monitored using near-infrared region II (NIR-II) imaging technology. The results demonstrated that the platelet membrane-coated biomimetic vesicles exhibited a notable increase in fluorescence signal intensity during the fresh thrombus targeting experiment, whereas other control groups exhibited weaker signals, thereby confirming the superior targeting capability of the platelet membrane-modified drug delivery system (Figure S8). Subsequently, the in vitro thrombolytic efficacy of the nanodrug delivery system was evaluated. The fresh thrombus samples were randomly divided into four groups (PBS, PBNZ, UK, and UK-PBNZ@PM) and treated accordingly. It is noteworthy that the UK-PBNZ@PM group demonstrated a markedly enhanced thrombolytic efficacy (43.16 ± 6.99 %) in comparison to the UK group (22.46 ± 9.50 %) at an identical urokinase (UK) concentration (100U/g). This finding indicates that the targeting effect of the platelet membrane facilitated the efficient accumulation of the UK-PBNZ@PM complex at the thrombus site, thereby significantly enhancing thrombolytic efficacy (Fig. 6b). In conclusion, the platelet membrane-modified biomimetic vesicular nanodrug delivery system developed in this study not only exhibits excellent targeting performance but also significantly enhances thrombolytic treatment efficiency, offering new strategies and insights for the treatment of thrombotic disorders.

Building upon previous research, we conducted a more comprehensive investigation into the thrombolytic efficacy of the UK-PBNZ@PM nanodrug delivery system in vivo. To this end, we induced thrombus formation in the left abdominal superficial epigastric vein of SD rats using a 10 % ferric chloride solution and randomly divided the rats into three groups (n = 3), administering tail vein injections with phosphate-buffered saline (PBS), UK (100U/g), and the UK-PBNZ@PM (UK: 100U/g). After a 24-h period, the rats were euthanized, and the relevant vascular samples were collected for histopathological analysis. To quantify the thrombolytic effect, the occluded area was divided by the total vessel area for each group. The findings demonstrated that the vessels in the PBS group exhibited near-complete occlusion, whereas the UK group demonstrated a significant reduction in the occluded area, reaching 52.03 % ± 10.4 %. It is noteworthy that the UK-PBNZ@PM group exhibited a further reduction in the occluded area, reaching 29.19 % ± 2.45 % (Fig. 6c and d). This evident data comparison to illustrate the superior thrombolytic efficacy of the biomimetic vesicular nanodrug delivery system in comparison to UK alone. To illustrate the thrombolytic process visually, we initially utilized an ICG contrast agent to visualize the vessels, followed by the administration of the pharmaceutical agents. Subsequently, thrombus formation was induced with ferric chloride, resulting in a notable reduction in fluorescence signal at the thrombus site in the left abdominal superficial epigastric vein of the rats. Six hours after the administration of the pharmaceutical agent, the ICG contrast agent was re-administered for imaging purposes. The results demonstrated that the shadow length at the thrombus site exhibited a notable increase in the PBS group, whereas the UK group demonstrated some improvement, although a visible shadow remained. In contrast, the UK-PBNZ@PM group exhibited near-complete vessel patency with no discernible low-intensity regions (Fig. 6e). In conclusion, our study not only corroborates the exceptional targeting and thrombolytic capabilities of the UK-PBNZ@PM nanodrug delivery system in vitro but also demonstrates its equally impressive performance in vivo. This discovery presents a novel and efficacious solution for the treatment of thrombotic disorders.

### 3.5 UK-PBNZ@PM exhibits exceptional biocompatibility in vivo

**Fig. 7:**
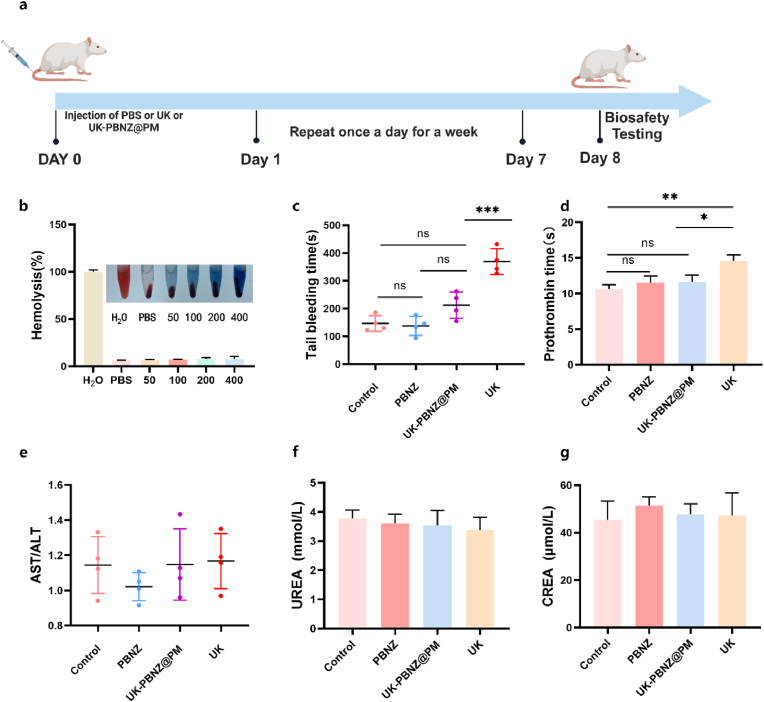
(a) Schematic diagram of the biological safety assessment process. PBS (100 μL), UK (100 U/g), UK-PBNZ@PM and PBNZ (1.36 mg/ml, 100 μL) were injected into the tail vein for one week. (b) Blood compatibility was tested by incubating blood with H2O, saline, PBS and PBNZ (50, 100, 200, 400 μg/ml). (c) The bleeding time in the tail bleeding assay. (d) Prothrombin time. (e) Biochemical indicators of liver function (AST: glutamic oxaloacetic transaminase, ALT: glutamic pyruvic transaminase). (f–g) Biochemical indicators of renal function.

In this study, we sought to comprehensively evaluate the biological safety of UK-PBNZ@PM. To this end, we designed and conducted a series of in vivo experiments. As illustrated in Fig. 7a, a series of agents were initially administered via tail vein injection to SD rats. The agents consisted of PBS (100 μL, serving as the control group), PBNZ solution (1.36 mg/ml, 100 μL), UK solution (100 U/g, 100 μL), and the UK-PBNZ@PM (1.36 mg/ml, 100 μL). One week later, the bleeding time of rats in each group was determined through standard tail-cutting experiments. The results demonstrated that, in comparison to the control group, both the PBNZ and UK-PBNZ@PM groups exhibited bleeding times within the normal range. Conversely, rats that received UK alone exhibited a significantly prolonged bleeding time (Fig. 7c). These results suggest that UK administration alone may pose a potential risk of bleeding. Subsequently, the experimental animals were euthanized, and blood samples were collected for a series of hemocompatibility and biochemical indicator analyses. The results of the hemolysis test (Fig. 7b) clearly demonstrated the excellent hemocompatibility of the PBNZ material, indicating that it does not cause red blood cell rupture during blood circulation, thereby safeguarding the stability of the blood system. Additional coagulation function tests demonstrated the capacity of the UK-PBNZ@PM to mitigate the risk of bleeding. In particular, the coagulation time of rats injected with UK was markedly prolonged to 14.57 ± 0.85 s, whereas the coagulation time of rats in the UK-PBNZ@PM group, which received the same dose of UK but encapsulated in PM membranes, was shortened to 11.63 ± 0.95 s (Fig. 7d). This discovery not only corroborates the efficacy of membrane modification technology in controlling the release rate of UK, but also suggests its beneficial role in reducing bleeding complications during thrombolytic therapy. Afterwards, biochemical tests were performed on the liver and kidney functions of rats in each group, and the results showed that there were significant differences in the above 667 indices among the groups (Fig. 7e and f). Finally, the main organs were fixed with formaldehyde and embedded in paraffin for safety assessment. No organ tissue lesions were observed in HE sections (Figure S10). These findings provide compelling evidence that the bionic vesicle (UK-PBNZ@PM) is safe for vital organs in vivo, eliminating the possibility of systemic toxic reactions. In conclusion, the results of this study, which employed a systematic approach to in vivo experimentation, demonstrate that the UK-PBNZ@PM complex exhibits not only excellent biological safety but also effectively reduces bleeding risks by controlling the release rate of thrombolytic drugs. These findings provide a safer and more effective strategy for thrombolytic therapy.

### 3.6 UK-PBNZ@PM enhance flap survival rates by maintaining microenvironmental homeostasis in ischemia-reperfusion injury

**Fig. 8:**
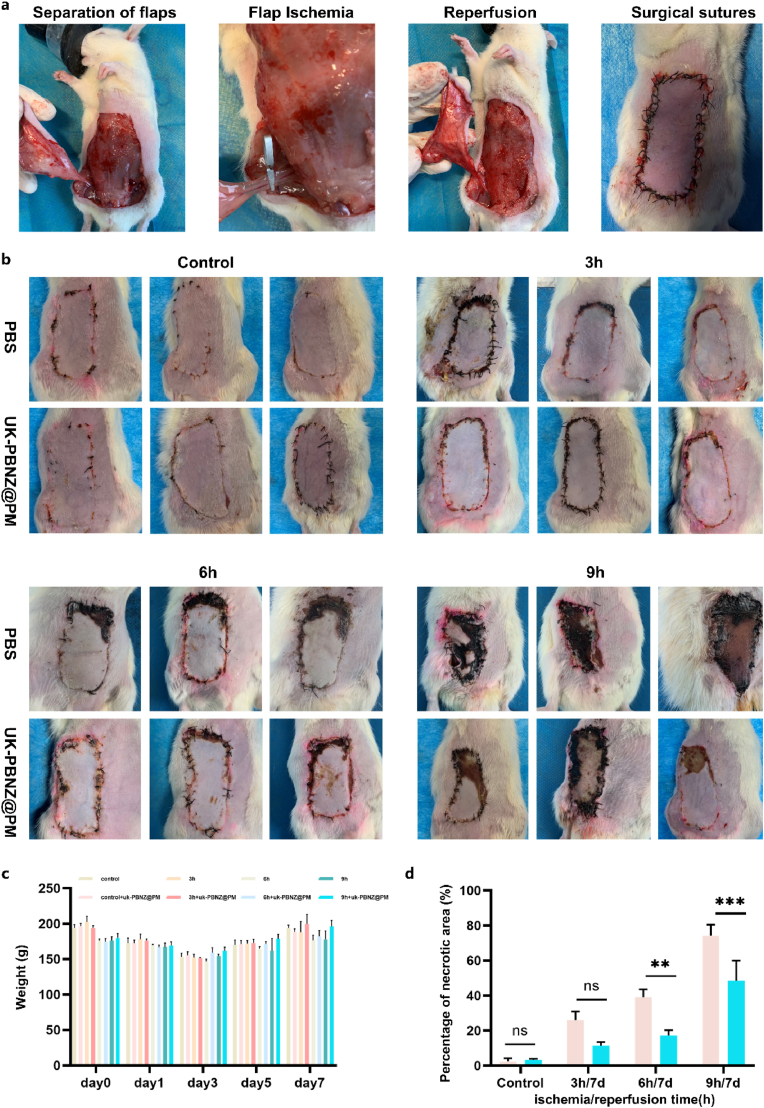
Establishment of a rat flap ischemia-reperfusion injury model. (a) Establishment of a rat flap ischemia-reperfusion injury model. (b) Morphological changes of the flap after one week with or without UK-PBNZ@PM treatment at different times (3h, 6h, and 9h) of flap ischemia. (c) body weight changes of rats within one week after surgery. (d) Image j quantitative analysis of the area of flap necrosis in rats. (n = 3; ∗∗: p < 0.01; ∗∗∗: p < 0.001; ns: no significant difference).

In this study, based on previous in vitro experimental results, we have confirmed that the PBNZ material exhibits remarkable capabilities in ROS scavenging, anti-inflammatory effects, and anti-apoptotic properties. The bionic vesicle (UK-PBNZ@PM), formed by loading UK onto PBNZ and further modifying it with PM, not only retains the above advantages but also demonstrates highly efficient targeted thrombolytic abilities, displaying significant therapeutic and preventive effects against both high-risk postoperative thrombosis and early thrombosis. To validate the therapeutic efficacy of the bionic vesicle in vivo, we conducted a comprehensive investigation using a rat skin flap model. The experimental design involved the utilization of the left abdominal wall anterior circumflex artery and vein of rats as the blood supply, with the preparation of skin flaps measuring 3 cm × 6 cm in size (as illustrated in Fig. 8a). Considering the research findings of Chin-Yu Yang and others, it became evident that lymphatic drainage and vascularized lymph node perfusion in rodents are significantly influenced by ischemia duration. Furthermore, preliminary studies indicated that different ischemia durations have differential impacts on cell viability and mitochondrial damage. With this understanding, the experimental rats were randomly divided into four groups, each consisting of six rats: a sham surgery group, a 3h ischemia group, a 6h ischemia group, and a 9h ischemia group. Within each group, three rats were randomly assigned to receive the UK-PBNZ@PM (1.36 mg/100 g) and the UK treatment, while the remaining three rats were injected with PBS as a control. Observations were conducted on a continuous basis over the course of one week. The experimental results are shown in Fig. 8b–d and S11-13. As the ischemic time increased, the necrosis area of the flap gradually expanded. The necrosis areas of the flap in the 3h, 6h, and 9h ischemic groups were 25.97 ± 4.88 %, 39.12 ± 4.44 %, 74.26 ± 6.20 %. It is noteworthy that the necrosis area of the skin flaps in rats treated with UK-PBNZ@PM was significantly reduced compared with that in the PBS control group, which were 11.36 ± 2.09 %, 17.26 ± 3.04 % and 48.46 ± 11.52 %, respectively. At 3h and 6h, the therapeutic effect was not significantly different from that of biomimetic vesicles, but at 9h, the therapeutic effect of UK was not ideal. We speculate that this may be because UK has no ability to scavenge free radicals, resulting in the therapeutic effect becoming worse as the ischemia time increases. In conclusion, the present study employed the construction of a rat skin flap model to conduct a comprehensive evaluation of the therapeutic effects of the bionic vesicle in vivo. The results validate the highly efficient thrombolytic capabilities of the bionic vesicle and highlight its remarkable advantages in reducing tissue damage and promoting recovery. They provide a novel and promising therapeutic strategy for clinical antithrombotic treatment.

**Fig. 9:**
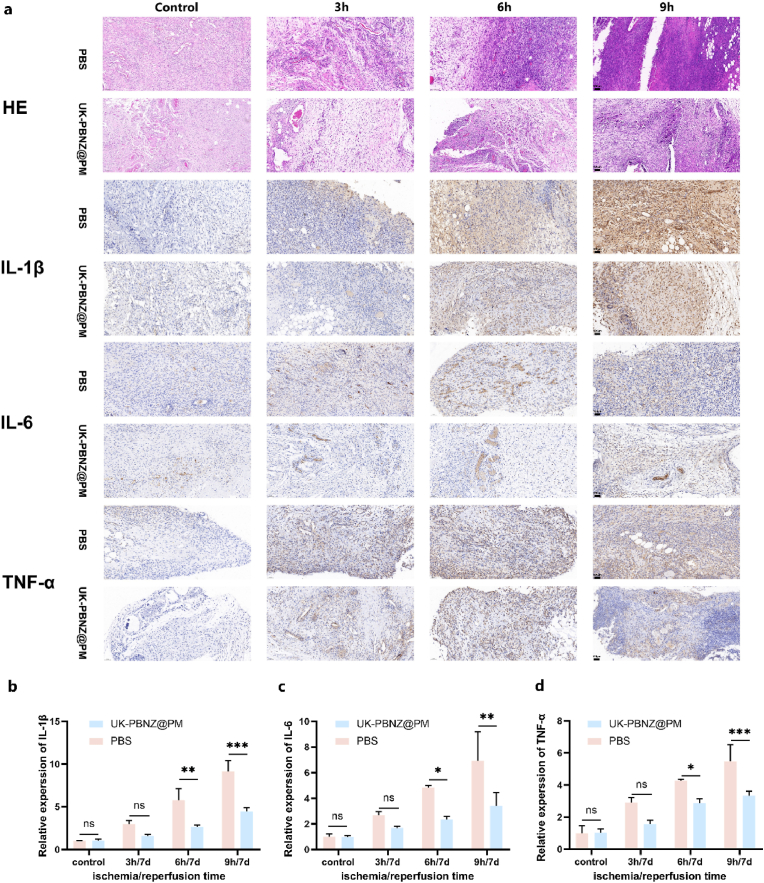
Histological analysis of the ischemia-reperfusion flap tissue. (a) H&E staining and immunohistochemical detection of IL-1β, IL-6 and TNF-α expression; scale bar = 100 μm. Image J quantitative analysis of the relative expression rates of positive area for IL-1β (b), IL-6 (c) and TNF-α (d). Homogenization was done with control positive area.

**Fig. 10:**
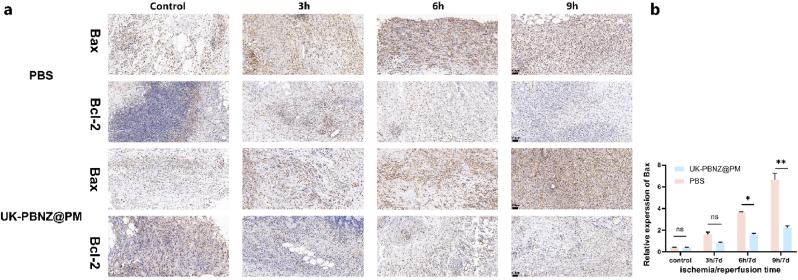
Histological expression of apoptosis proteins in flap ischemia-reperfusion injury. (a) Immunohistochemical detection of Bax and Bcl-2 expression; scale bar = 100 μm. (b) Image J quantitative analysis of positive expression of Bax protein relative to Bcl-2 protein.

To gain further insight into the specific protective mechanisms of UK-PBNZ@PM in vivo, this study employed a precise sampling technique, extracting tissue blocks of 0.5 cm × 0.5 cm from identical locations on the skin flaps of rats in each group. These samples were then subjected to detailed immunohistochemical analyses, focusing on key inflammatory factors such as TNF-α, IL-1β, and IL-6. The results demonstrated that in the control group that did not receive UK-PBNZ@PM treatment, the positive expression rates of these inflammatory factors exhibited a gradual increase with the extension of flap ischemia time, reaching a peak in the 9h ischemia group. In contrast, in the ischemic 3h and 6h groups, the positive expression rates of inflammatory factors in the skin flaps of rats injected with UK and UK-PBNZ@PM were significantly reduced, but in the ischemic 9h group, TNF-α and IL-6 in the UK group were significantly decreased. - 6 had a slightly higher positive expression rate, while the inflammatory factors in the UK-PBNZ@PM group all showed a significant decrease, which was highly consistent with the anti-inflammatory ability shown in vitro (as shown in Fig. 9a–d, S14-S17). Moreover, the relative expression of Bax and Bcl-2 proteins within the apoptotic pathway was validated in tissue samples. The results demonstrated that as the duration of ischemia increased, the positive expression rates of Bax and Bcl-2 proteins also elevated, indicating an enhanced activation of the apoptotic pathway. However, following treatment with UK-PBNZ@PM and UK, we observed a decline in the relative expression levels of the Bax protein. Yet, within the 9-h treatment group, the reduction seen in the UK-treated subset was notably less pronounced when compared to the overall trend within the 9-h group (as illustrated in Fig. 10a, b, S18, and S19). This observation aligns well with the trends we had previously noted in our Western blot analysis of the in vitro model of glucose-oxygen deprivation. Based on the comprehensive analysis, it can be concluded that UK-PBNZ@PM effectively mitigates the expression of inflammatory factors in vivo, thereby alleviating inflammatory responses and maintaining the homeostasis of the intravascular environment. Furthermore, the bionic vesicle inhibits the activation of apoptotic pathways, thereby reducing the occurrence of cellular apoptosis. These two mechanisms act in concert to markedly reduce ischemia-reperfusion injury in skin flaps, enhance their protection and repair, and significantly increase their survival area. These findings not only enhance our comprehension of the biological activities of UK-PBNZ@PM but also furnish a robust experimental foundation and theoretical basis for the prospective development of innovative pharmaceutical agents against ischemia-reperfusion injury.

## 4 Discussion and conclusion

Flap transplantation represents a cornerstone of tissue repair and reconstruction in plastic surgery, particularly in head and neck surgery. Its widespread application and remarkable outcomes offer hope for numerous patients seeking to reshape their lives. Nevertheless, despite remarkable technological advancements, postoperative complications such as thrombosis and ischemia-reperfusion (I/R) injury continue to pose a significant barrier to the success of flap transplantation [45,46]. Statistical data indicates that approximately 15 % of transferred flaps necessitate recanalization procedures and intricate postoperative revisions due to these complications, emphasizing the constraints of contemporary therapeutic strategies in preventing and managing such complications [47]. It is particularly noteworthy that the scarcity of early diagnostic and therapeutic modalities post-flap surgery often results in the identification of flap crises when vascular injury and thrombosis have already progressed to an irreversible stage, compelling clinicians to rely on high-risk secondary surgical interventions. Unfortunately, the results of these secondary surgeries are frequently unsatisfactory, posing significant challenges to the survival of the flap and the restoration of function. Furthermore, individual patient characteristics, including age, extent of the lesion, and overall health status, result in significant variability in surgical duration. This is an area that current clinical thrombolytic treatments are unable to adequately address, due to a lack of precision in individualized treatment strategies [48]. In view of these challenges, we have devised a series of models with varying ischemia durations, with the objective of elucidating the specific impact of surgical duration on flap necrosis extent and survival area. This study seeks to elucidate the fundamental pathophysiological mechanisms and provide insights that will inform the development of novel therapeutic approaches. By conducting a series of experiments, we have not only validated ischemia time as a crucial factor affecting flap prognosis, but more importantly, based on this finding, we have proposed an innovative ischemia-time-oriented, personalized flap salvage treatment strategy. This strategy has the potential to equip clinicians with more scientific and precise decision-making tools, enabling them effective interventions at an early stage to mitigate flap necrosis risks, thereby enhancing the overall success rate of flap transplantation and improving patients' quality of life.

In the field of plastic surgery, the occurrence of vascular complications following flap transplantation, particularly thrombosis, continues to be a significant factor influencing the success of surgical procedures. At present, drug thrombolysis (e.g. urokinase, tPA) and surgical embolectomy represent the prevailing treatment modalities, offering some degree of relief yet with notable limitations. The efficacy of drug thrombolysis is limited by its the brief half-life in the systemic circulation, which makes it challenging to maintain a sustained and effective thrombolytic effect. Furthermore, the administration of drugs systemically can precipitate severe bleeding complications, such as intracranial hemorrhage, which significantly increases the risks associated with treatment [49,50]. Conversely, while mechanical embolectomy represents a direct approach, its success rate and efficacy remain inconsistent [51]. Additionally, the reperfusion process itself can inflict secondary damage on the flap and vessels. Considering these challenges, this study concentrates on the crucial elements of thrombolytic therapy, namely ischemia time and internal environment regulation. We propose a comprehensive therapeutic strategy that employs biomimetic vesicles UK-PBNZ@PM in an innovative manner. These biomimetic vesicles ingeniously integrate the thrombolytic drug UK with the protective component PBNZ, with the aim of overcoming the limitations of traditional thrombolytic therapies through precise delivery and synergistic effects. In particular, the UK-PBNZ@PM approach reduces the dosage of UK, thereby mitigating the adverse effects associated with the drug. At the same time, it exploits the properties of PBNZ to effectively attenuate high levels of reactive oxygen species (ROS) and the inflammatory environment surrounding the thrombus, thereby significantly reducing the extent of reperfusion injury. By means of precise regulation by biomimetic vesicles, a complete sequential treatment regimen is achieved. This strategy not only enhances thrombolytic efficiency and safety but also demonstrates significant advantages in preventing reperfusion injury, offering a novel perspective and a powerful tool for salvaging ischemic flaps. The experimental results indicate that the thrombolytic effect on skin flaps treated with UK-PBNZ@PM is significantly enhanced compared to those treated with thrombolytic drugs alone. Moreover, no notable complications of bleeding or reperfusion injury were observed. This research not only paves the way for novel approaches to treating vascular complications following flap transplantation but also offers insights and inspiration for thrombolytic strategies in other related fields. In the future, with further investigation and enhancement of biomimetic vesicle technology, we are confident that this innovative strategy will play a pivotal role in broader medical practices, offering safer and more effective treatment options for patients.

In the pursuit of efficacious and safe thrombolytic treatment strategies, the safety, effectiveness, and clinical translation rate of thrombolytic therapies have emerged as core metrics for evaluating the efficacy and practicality of treatment methods. The introduction of an innovative platelet membrane-coated nanozyme drug delivery system has not only overcome numerous challenges faced by traditional thrombolytic therapies but has also achieved remarkable progress in enhancing treatment safety and effectiveness. This system exploits the intrinsic camouflage properties of platelet membranes to effectively evade recognition and elimination by the immune system, thereby enabling stable circulation and precise delivery of drugs within the body. Furthermore, the targeting function of platelet membranes facilitates the accumulation of thrombolytic drugs and nanozymes at sites of thrombosis or injury, thereby reducing the systemic dosage of thrombolytic drugs and minimizing the risk of bleeding and other adverse effects. This ensures the safety of thrombolytic treatment. Moreover, the biomimetic vesicle nanodrug delivery system employed in this study has a robust clinical history and exhibits excellent biosafety for each of its components. Prussian blue, a widely utilized anti-radiation medication in clinical settings, has demonstrated its safety and efficacy [52,53]. Urokinase, a primary thrombolytic therapy agent and an endogenous enzyme present in the human body, is non-immunogenic and safe for use. Furthermore, the platelet membrane containing targeting peptides can be directly extracted from the patient's blood, further enhancing the system's personalization and biocompatibility. The clinical application backgrounds of these components not only provide robust support for the safety of the biomimetic vesicle nanodrug delivery system but also indicate significant potential for clinical translation. In the future, with further research and technological advancements, this system is likely to become a significant breakthrough in the field of thrombolytic treatment, offering safer and more effective treatment options for numerous patients and driving progress and development in clinical practice.

This study examined the pivotal role of ischemia time, a critical determinant of skin flap survival following transplantation. The findings revealed a pronounced influence of varying ischemia durations on flap prognosis. The experimental results indicated a significant linear increase in both the necrotic area and necrosis rate of the flaps as ischemia time was prolonged (from 3 h to 9 h). It is noteworthy that when ischemia time reached 6 h, large-scale necrosis began to manifest in the flaps. By 9 h, the survival area of the flaps has nearly collapsed, with even pharmacological interventions failing to salvage the majority of the necrotic tissues. However, in the group with a shorter ischemia time of 3 h, we observed relatively mild apoptosis in vitro and necrosis in vivo. This finding provides a valuable reference time window for clinical treatment, suggesting that when ischemia time exceeds 3 h, close monitoring and preventive treatment of the flaps should be prioritized to minimize necrosis risks. Although this study has begun elucidate the relationship between ischemia time and flap survival rate, numerous questions remain regarding the specific changes that occur within each time segment. For example, further investigation is required to determine whether a buffer period exists between 3 and 6 h and to identify the precise threshold for the onset of extensive necrosis between 6 and 9 h. Furthermore, individual differences in ischemia tolerance among rats due to constitutional variations manifest as personalized characteristics, necessitating a more nuanced consideration of the impact of individual variability on experimental results in future studies. Notwithstanding these limitations, the current findings underscore the indispensable role of ischemia time in guiding clinical treatment. These findings highlight the importance of timely assessment of ischemia time and subsequent measures post-skin flap transplantation, as they are crucial for enhancing flap survival rates. Going forward, we will continue to deepen our research in this field, employing more refined time divisions and comprehensive individual difference analyses to provide more precise and scientific guidance for treatment strategies following skin flap transplantation.

This study successfully developed a biomimetic nanoparticle, UK-PBNZ@PM, based on an ischemia-reperfusion (I/R) model with varying ischemia durations. The nanoparticle integrates sequential thrombolysis and prevention of ischemia-reperfusion injury, representing a significant advancement in the treatment of ischemic injury. This innovative therapeutic approach demonstrates remarkable efficacy through multiple mechanisms, thereby opening new avenues for the treatment of complications arising from skin flap transplantation. Firstly, the ingenious modification with platelet membranes endows UK-PBNZ@PM with exceptional thrombus-targeting ability, significantly enhancing the delivery efficiency and local concentration of thrombolytic drugs. This feature has the dual benefit of reducing systemic drug exposure and the risk of systemic side effects, while also ensuring that the thrombolytic effect is directly targeted at the lesion, thereby improving treatment outcomes. Secondly, the incorporation of urokinase (UK), an endogenous enzyme naturally present in the body, ensures the biosafety of the treatment process. The non-immunogenic properties of UK-PBNZ@PM permit its stable existence in the systemic circulation, wherein it is not rapidly cleared by the immune system. This provides a guarantee for continuous and effective thrombolytic therapy. Moreover, the incorporation of Prussian blue nanoparticles (PBNZ) endows the biomimetic nanoparticle with robust ROS scavenging and anti-inflammatory capabilities. During reperfusion, PBNZ effectively neutralizes excess reactive oxygen species (ROS), thereby mitigating inflammatory responses and significantly reducing the extent of ischemia-reperfusion injury. This dual mechanism not only safeguards the structural integrity of vascular walls and surrounding tissues but also expedites flap recovery. It is of note that UK-PBNZ@PM displays considerable promise regarding its clinical translation potential. The individual components of the treatment have already been validated and applied in clinical settings, thereby establishing a robust foundation for future clinical trials and applications. Moreover, through comprehensive investigations employing in vitro and in vivo models with varying ischemia durations, we offer invaluable insights that can inform clinical practice. The results demonstrate that the biomimetic nanoparticle exerts substantial therapeutic effects even under shorter ischemia times, while its sequential treatment advantage becomes more pronounced with longer ischemia durations. This provides a scientific foundation for emergency interventions following skin flap transplantation. Ultimately, the therapeutic outcomes in rat flap models serve to corroborate the efficacy of UK-PBNZ@PM in preventing ischemia-reperfusion injury, particularly in terms of its precise targeted thrombolysis and sequential treatment effects. This innovative therapy has been demonstrated to significantly improve flap survival rates and functional recovery, while also establishing a new benchmark for the treatment of vascular complications in the field of plastic surgery. In the future, we anticipate further optimization and in-depth research to expand the application of this therapy, thereby extending benefits to a broader patient population.

## CRediT authorship contribution statement

**Linzhong Yang:** Writing – original draft, Visualization, Validation, Software, Methodology, Investigation, Formal analysis, Data curation, Conceptualization. **Yuanchen Liu:** Writing – original draft, Visualization, Validation, Software, Methodology, Investigation, Formal analysis, Data curation, Conceptualization. **Cheng Tao:** Writing – original draft, Visualization, Validation, Software, Methodology, Investigation, Formal analysis, Data curation, Conceptualization. **Zichen Cao:** Writing – review & editing, Software, Methodology, Data curation. **Shilin Guo:** Writing – review & editing, Formal analysis. **Zheng Wei:** Writing – review & editing, Funding acquisition. **Yanyi Wang:** Writing – review & editing. **Tao Liu:** Writing – review & editing. **Lin Chen:** Writing – review & editing, Formal analysis, Data curation. **Ke Xiong:** Writing – review & editing, Methodology. **Xingyu Luo:** Writing – review & editing, Funding acquisition. **Jianchuan Ran:** Writing – review & editing, Resources, Project administration, Funding acquisition. **Wei Han:** Writing – review & editing, Resources, Project administration, Funding acquisition.

## Consent to participate

Originality of Content: The manuscript submitted is an original work of authorship and has not been previously published, either in whole or in part, in any form or medium, including electronically. The manuscript is not being considered for publication elsewhere.

Authorship Responsibility: We have all contributed significantly to the intellectual content of the manuscript and have taken due care to ensure the integrity of the work. We acknowledge that all authors are listed and that no one who contributed significantly to the work has been omitted.

Approval of Final Version: We have seen and approved the final version of the manuscript, which we believe accurately reflects our contributions and the results of our research.

Responsibility for Content: We assume full responsibility for the content and data presented in the manuscript. We confirm that any claims made in the manuscript are supported by appropriate citations and data.

Compliance with Policies: We are aware of and comply with any relevant policies or ethical guidelines governing authorship, data reporting, and the use of images or human subjects in our research.

Permission for Publication: We grant permission for the manuscript to be published in the journal, subject to the journal's editorial policies and processes.

Consequences of Non-Compliance: We understand that failure to comply with these terms may result in the withdrawal of the manuscript from consideration for publication or the retraction of the article if already published.

We confirm our agreement to the above terms and conditions.

Linzhong Yang, Yuanchen Liu, Cheng Tao, Zichen Cao, Shilin Guo, Zheng Wei, Yanyi Wang, Tao Liu, Lin Chen, Ke Xiong, Xingyu Luo, Jianchuan Ran, Wei Han.

## Ethics approval

The animal experiments have received preapproval from the Institutional Animal Care and Use Committee (IACUC) of the Medical School of Nanjing University (IACUC—D2304022). All mice were housed in SPF-grade animal facilities.

## Availability of data and materials

The data and materials are available for consultation upon request.

## Declaration of competing interest

The authors declare that they have no known competing financial interests or personal relationships that could have appeared to influence the work reported in this paper.

## Data Availability

Data will be made available on request.
