# Supplementary material for: Bionic nanovesicles sequentially treat flaps with different durations of ischemia by thrombolysis and prevention of ischemia-reperfusion injury
Source: Mater Today Bio. 2025 Jan 30;31:101529. doi: 10.1016/j.mtbio.2025.101529 (PMC11846944; doi:10.1016/j.mtbio.2025.101529)
Supplement: Multimedia component 1 [file mmc1.docx]

Supplementary materials


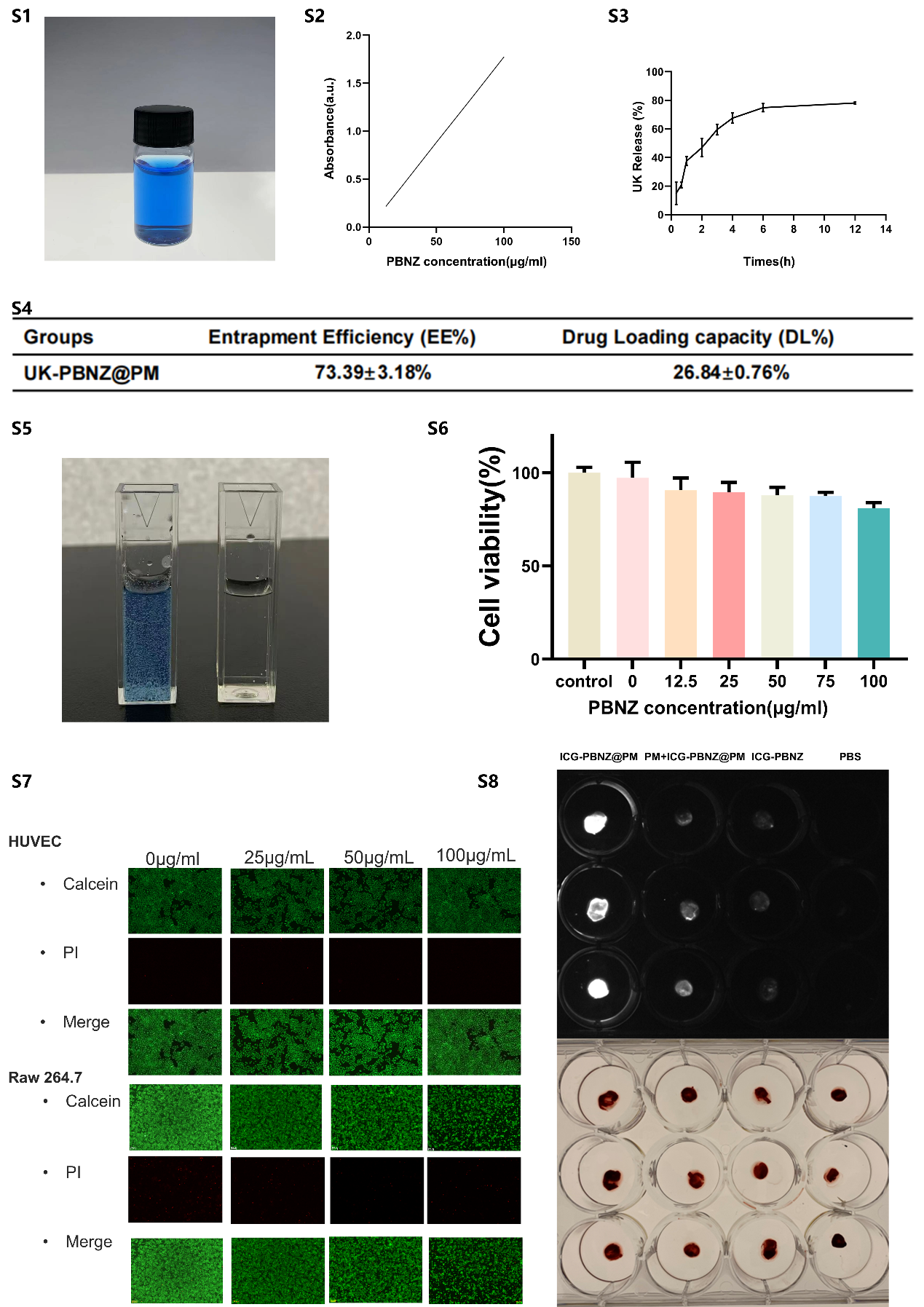


Supplementary materials: (S1) Aqueous solution of PBNZ. (S2) Standard concentration curve of PBNZ. (S3) Drug release curve of UK-PBNZ@PM (20min 40min 1h 2h 3h 4h 6h 12h). (S4) Encapsulation rate and drug loading. (S5) UK-PBNZ@PM was incubated with hydrogen peroxide at room temperature for 5 minutes, producing many bubbles. (S6) CCK-8 assay of RAW 264.7 cell viability after incubation with different concentrations of PBNZ for 24 hours. (S7) RAW264.7 and HUVECs cells were incubated with PBNZ for 24 hours, and the cells were stained for live and dead cells. Scale bar=100nm. (S8) Thrombus imaging after co-incubation with ICG-PBNZ@PM, PM+ICG-PBNZ@PM, ICG-PBNZ, and PBS, respectively.


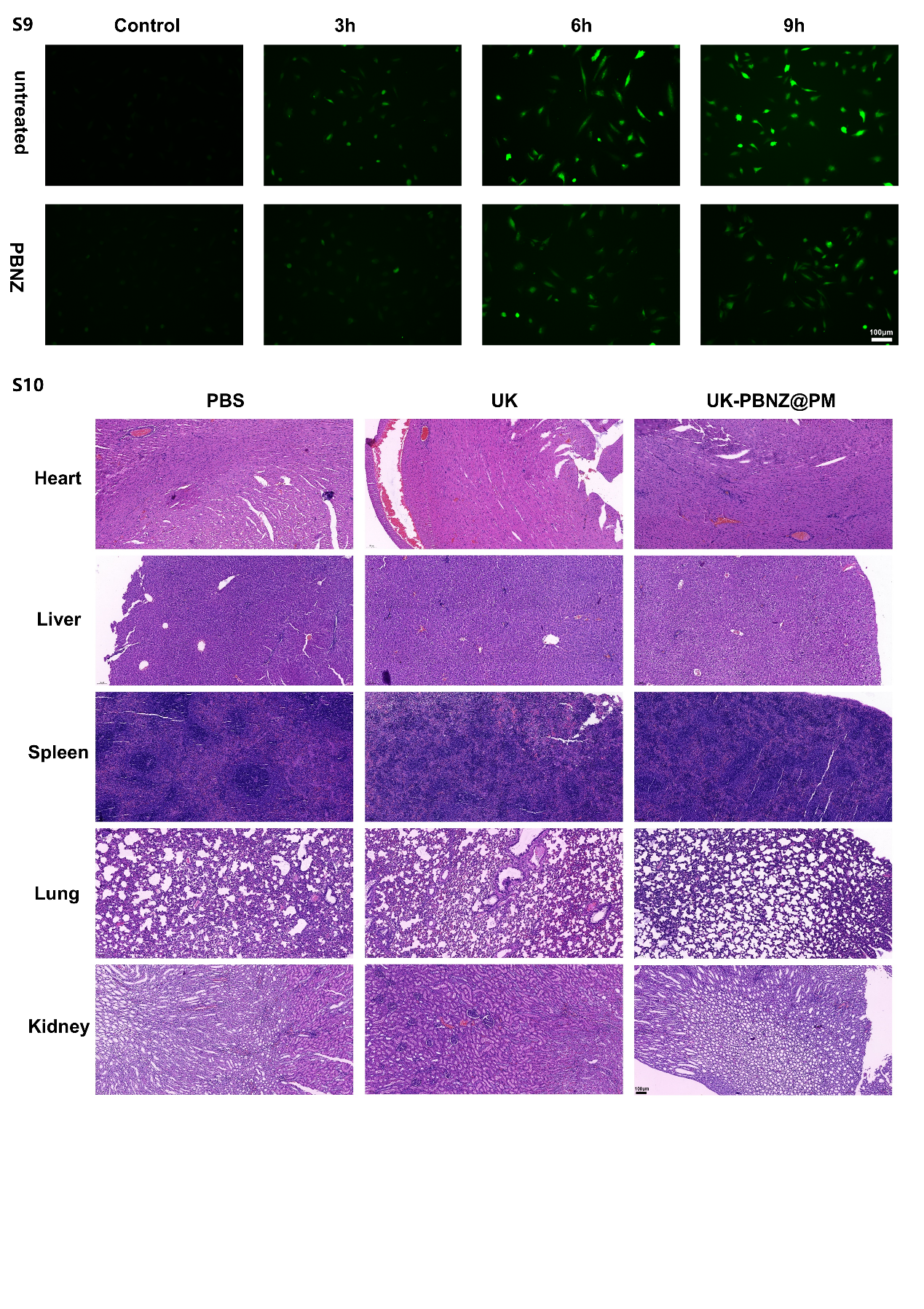


Supplementary materials: (S9) Fluorescence microscopy revealed that PBNZ treatment reduced the level of ROS in HUVEC cells in the glucose-oxygen deprivation model. Scale bar: 100μm. (S10) HE staining of main organs. Scale bar=100μm.


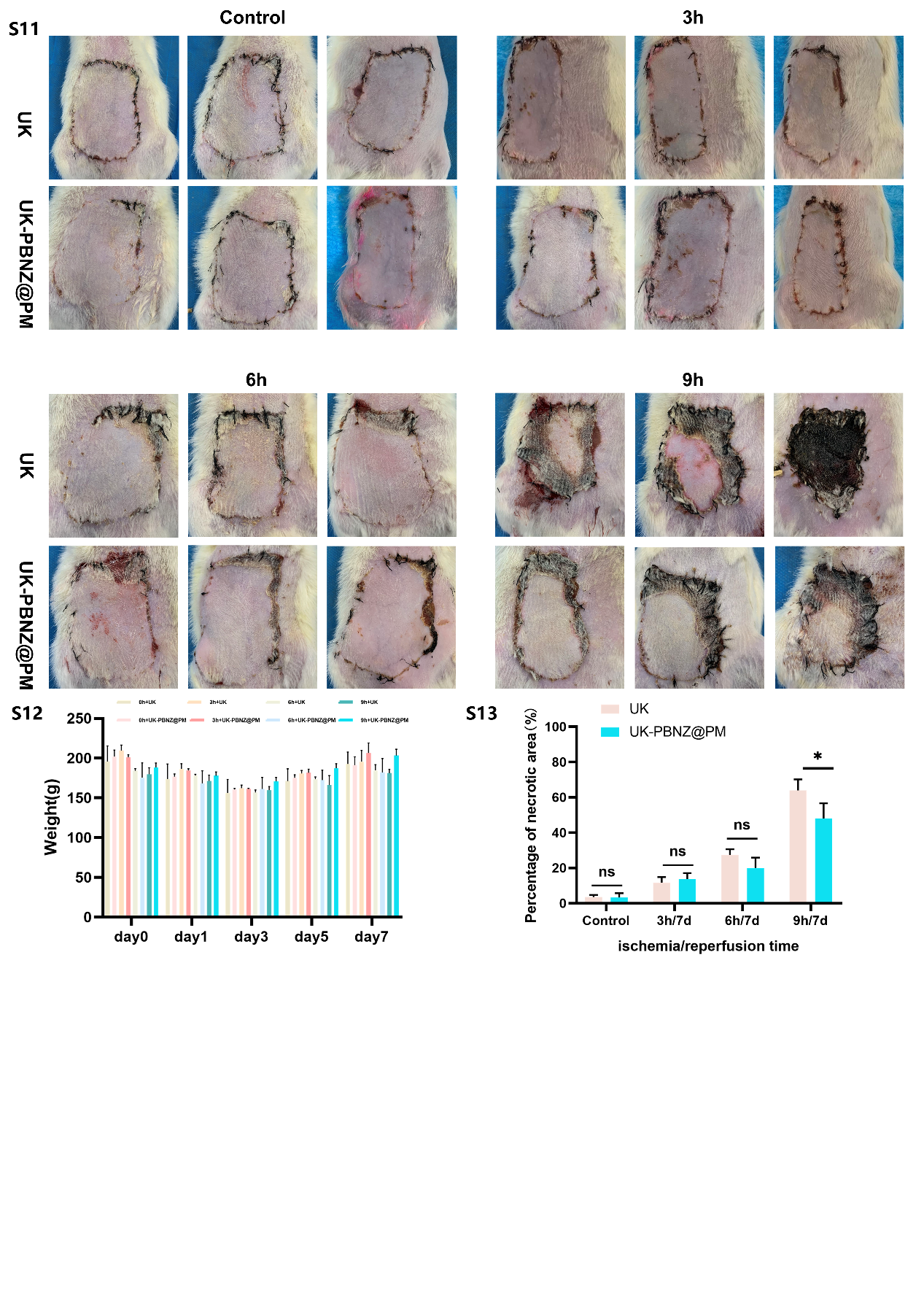


Supplementary materials: (S11) Morphological changes of the flap after treatment with UK or UK-PBNZ@PM for one week at different time points (3h, 6h, 9h) of flap ischemia. (S12) Body weight changes of rats within one week after surgery. (S13) Image J quantitative analysis of the necrotic area of rat skin flap. (n=3; * p < 0.05; ns: no significant difference).


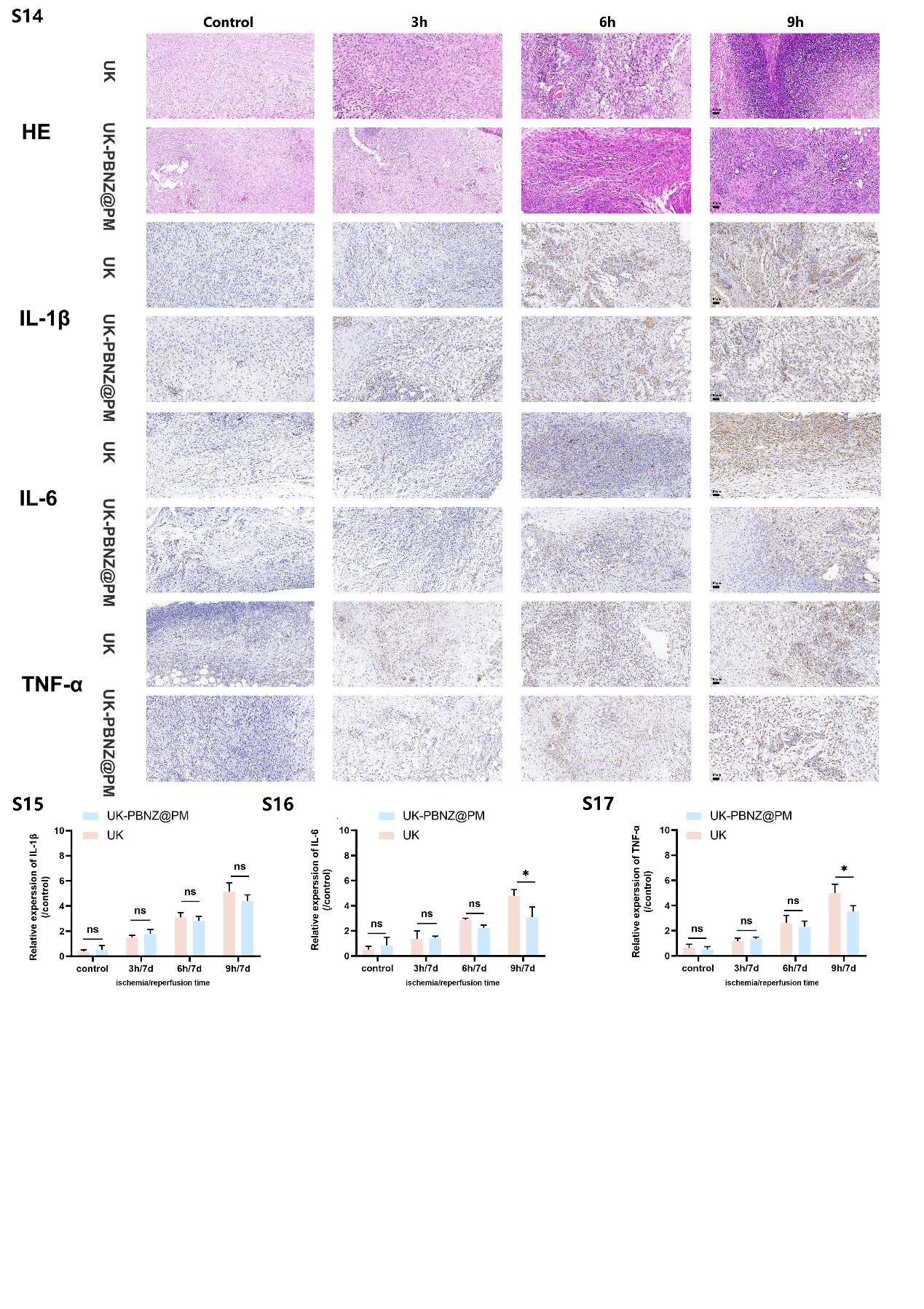


Supplementary materials: (S14) H&E staining and immunohistochemical detection of IL-1β, IL-6 and TNF-α expression; scale bar = 100μm. Image J quantitative analysis of the relative expression rates of positive area for IL-1β (S15), IL-6 (S16) and TNF-α (S17). Homogenization was done with control positive area. (n=3; * p < 0.05; ns: no significant difference).


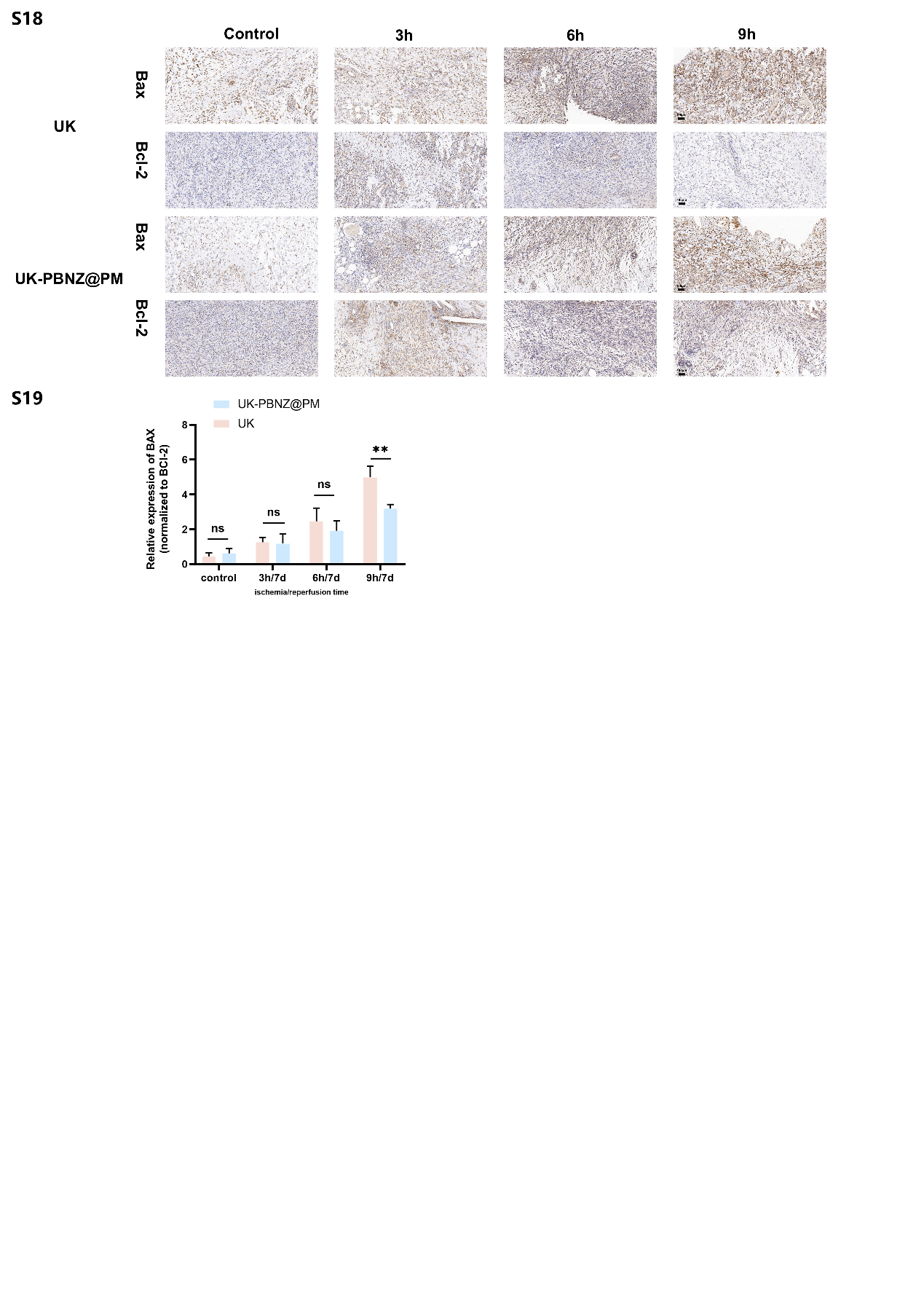


Supplementary materials: (S18) Immunohistochemical detection of Bax and Bcl-2 expression; scale bar = 100μm. (S19) Image J quantitative analysis of positive expression of Bax protein relative to Bcl-2 protein. (n=3; ** p < 0.01; ns: no significant difference).
